# Preparing Workplaces for Digital Transformation: An Integrative Review and Framework of Multi-Level Factors

**DOI:** 10.3389/fpsyg.2021.620766

**Published:** 2021-03-23

**Authors:** Brigid Trenerry, Samuel Chng, Yang Wang, Zainal Shah Suhaila, Sun Sun Lim, Han Yu Lu, Peng Ho Oh

**Affiliations:** ^1^Lee Kuan Yew Centre for Innovative Cities, Singapore University of Technology and Design, Singapore, Singapore; ^2^Humanities, Arts and Social Sciences, Singapore University of Technology and Design, Singapore, Singapore

**Keywords:** digital transformation, digital disruption, digital technology, workplace, organization, employee, literature review, multi-level framework

## Abstract

The rapid advancement of new digital technologies, such as smart technology, artificial intelligence (AI) and automation, robotics, cloud computing, and the Internet of Things (IoT), is fundamentally changing the nature of work and increasing concerns about the future of jobs and organizations. To keep pace with rapid disruption, companies need to update and transform business models to remain competitive. Meanwhile, the growth of advanced technologies is changing the types of skills and competencies needed in the workplace and demanded a shift in mindset among individuals, teams and organizations. The recent COVID-19 pandemic has accelerated digitalization trends, while heightening the importance of employee resilience and well-being in adapting to widespread job and technological disruption. Although digital transformation is a new and urgent imperative, there is a long trajectory of rigorous research that can readily be applied to grasp these emerging trends. Recent studies and reviews of digital transformation have primarily focused on the business and strategic levels, with only modest integration of employee-related factors. Our review article seeks to fill these critical gaps by identifying and consolidating key factors important for an organization’s overarching digital transformation. We reviewed studies across multiple disciplines and integrated the findings into a multi-level framework. At the individual level, we propose five overarching factors related to effective digital transformation among employees: technology adoption; perceptions and attitudes toward technological change; skills and training; workplace resilience and adaptability, and work-related wellbeing. At the group-level, we identified three factors necessary for digital transformation: team communication and collaboration; workplace relationships and team identification, and team adaptability and resilience. Finally, at the organizational-level, we proposed three factors for digital transformation: leadership; human resources, and organizational culture/climate. Our review of the literature confirms that multi-level factors are important when planning for and embarking on digital transformation, thereby providing a framework for future research and practice.

## Introduction

The rapid advancement of digital technologies such as smart technology, artificial intelligence (AI) and automation, robotics, cloud computing, and the Internet of Things (IoT) is fundamentally changing the nature of work and organizations. Collectively termed the Fourth Industrial Revolution (Schwab, 2015) or Industry 4.0, the speed and scale of current technological change are raising concerns about the extent to which new technologies will radically transform workplaces or displace workers altogether (Acemoglu and Autor, 2011; Frey and Osborne, 2013; Brynjolfsson and McAfee, 2014). The impact of digital disruption on labor markets remains contested, with some predicting substantial job losses through automation within a short time period (Frey and Osborne, 2013; McKinsey and Company, 2017). Others paint a more optimistic picture, predicting that as many new jobs will be created by new technologies as are displaced (Arntz et al., 2017). Nonetheless, the effects of digitalization are already being felt across a number of job roles and industries (Skog et al., 2018) and it is clear that organizations need to integrate new technologies and transform business models to remain competitive (Sebastian et al., 2017). Despite significant academic attention on how digital technology is disrupting job tasks and occupations (e.g., Acemoglu and Autor, 2011; Brynjolfsson and McAfee, 2014), there is less understanding of how workers and organizations can best respond to disruptive technological change. A central concern is how to bolster employee and organizational resilience to disruption from new technologies.

Although digital transformation is a new and urgent imperative, there is a long trajectory of rigorous research across multiple disciplines that can readily be applied to grasp these emerging trends. The impact of technology in the workplace has been studied for several decades (Davis, 1989; Orlikowski, 1992) and has its origins in information systems, psychology, and sociology (Venkatesh et al., 2003), alongside contributions from organizational behavior, management and communications (Huber, 1990; Dewett and Jones, 2001; Orlikowski, 2010). Recently, there has been sharp increase in studies from business and strategic information systems (Matt et al., 2015; Hess et al., 2016), human resources (Bondarouk et al., 2017; Marler and Boudreau, 2017), and healthcare (Agarwal et al., 2010; Burton-Jones et al., 2020), suggesting that digital disruption is increasing in a wider variety of industries and occupations.

In light of the scope and scale of digital transformation we are currently witnessing and the wellspring of diverse and valuable academic perspectives that have emerged to make sense of these changes, we believe that an evidence review of relevant literature is especially timely. Furthermore, we seek to lend greater coherence to our overall understanding of this fast-evolving landscape by taking an integrative approach that seeks to draw linkages across different disciplinary approaches. Hence, we have reviewed studies across disciplines and organized their findings into a holistic, multi-level framework. Our framework identifies and consolidates key factors critical for an organization’s overarching digital transformation at the individual, group, and organizational levels.

### Key Dimensions of Digital Transformation

There is a clear business case for digital transformation. By integrating new technologies into strategic processes, digital transformation aims to change business operations, processes, and services (Matt et al., 2015; Hess et al., 2016). In turn, these new digital capabilities can improve performance and expand products, services and customer bases (Westerman et al., 2014; Verhoef et al., 2019), leading to increased sales and profits (Warner and Wäger, 2019). There is consensus that industry-leaders in innovation and digital transformation have a greater competitive advantage and can attract a wider range of customers and employees (Berman, 2012; Chanias et al., 2019). Moreover, organizations that are more responsive to market trends and can adapt quickly to customer demands will also have the “first choice of talent, partners and resources” (Berman, 2012, p. 22). Indeed, competing for skilled employees is often cited as a key challenge to industry and workforce digital transformation (Karacay, 2018). In this way, digital transformation is not only about technology (Kane et al., 2015) but requires a focus on employee factors, alongside shifts in organizational strategy, structures, and processes (Hess et al., 2016).

Digital transformation is a more recent academic concept, although it draws on previous theories of IT-enabled change (Besson and Rowe, 2012; Wessel et al., 2020). While digital transformation is similar to other organizational change processes (e.g., Orlikowski, 1992; Weick and Quinn, 1999), it is a distinct form of organizational change (Hess et al., 2016; Vial, 2019; Wessel et al., 2020). Studies of IT-enabled transformation have identified various factors in the change process, such as organizational inertia, process, agency, and performance (Venkatesh, 2000; Kim and Kankanhalli, 2009; Besson and Rowe, 2012). While prior theory on IT-enabled change can inform the study of digital transformation, recent research suggests that digital transformation is a process of deep, structural change that occurs through the integration of multiple technologies and fundamentally redefines organizational value and identity (Besson and Rowe, 2012; Skog et al., 2018; Wessel et al., 2020).

Defined as a process that “aims to improve an entity by triggering significant changes to its properties through combinations of information, computing, communication, and connectivity technologies” (Vial, 2019, p. 121), digital transformation can occur at the organizational or broader entity-level. However, in contrast to other forms of technological change, digital transformation differs in terms of its scale, speed, and scope (Matt et al., 2015; Hess et al., 2016). When viewed as a process, digital transformation includes three main stages (Verhoef et al., 2019). First, organizations go through digitization, which involves transferring processes and systems, such as paper-based or non-analog systems, into digital formats (Tekic and Koroteev, 2019). Next, digitalization entails further integration and optimization of digital technologies and IT-capabilities to improve processes and add value to existing operations and services (Verhoef et al., 2019). While the different phases of digitization, digitalization, and transformation often overlap, digital transformation is conceived as the final step in the process and is triggered by extensive digital capabilities (Verhoef et al., 2019).

Recent reviews have sought to integrate studies on digital transformation across different disciplines, contexts, and research streams (Hausberg et al., 2019; Vial, 2019) and identify different stages of digital transformation, including key strategies and requirements to facilitate transformation (Verhoef et al., 2019). Some have focused on digital work design and leadership (Cascio and Montealegre, 2016; Cortellazzo et al., 2019) as well as attention to human resource factors, such as the role of Human Resource Development (HRD) professionals in facilitating skills development due to technological change (Chuang and Graham, 2018; Ghislieri et al., 2018). Reviews of industry transformation in the context of manufacturing and Industry 4.0 have focused on process-model automation (Liao et al., 2017) although digital transformation is fast becoming a priority for many other industries. This shift is reflected in the literature, with recent studies and reviews focusing on digitalization and transformation in a range of industries (Chanias et al., 2019; Vial, 2019). Despite these helpful contributions, there has been less integration of how digital transformation impacts workers and organizations across multiple levels.

## Technology Acceptance and Perceptions and Attitudes Toward Technological Change

As organizations undergo digitalization and digital transformation, theories of technology acceptance provide important insights. With origins in information systems research and social psychology (Ajzen, 1985, 1991; Davis et al., 1989), several theoretical models exist to understand which factors influence a user’s decision to adopt a new technology or system. The Technology Acceptance Model (TAM) (Davis, 1989) is one of the most commonly used frameworks and implies that behavioral intention (BI) and attitudes predict technology usage in two key ways: the perceived usefulness (PU) of technology (i.e., the degree to which a person believes that a technology will be useful) and perceived ease-of use (PEOU) (i.e., the degree to which a person believes that using a particular technology will be easy to use). TAM has been extended (TAM2) to include subjective norms and system-specific technology use (Venkatesh, 2000; Venkatesh and Davis, 2000).

More recently, Venkatesh et al. (2003) proposed the Unified Theory of Acceptance and Use of Technology (UTAUT) that incorporated existing models with motivation (Davis et al., 1992; Vallerand, 1997), social cognitive theory (Bandura, 1986; Compeau and Higgins, 1995) and diffusion of innovations theory (Rogers, 1995). The UTAUT postulates that four key factors (i.e., performance expectancy, effort expectancy, social influence, and facilitating conditions) and four moderators (i.e., age, gender, experience, and voluntariness) predict technology adoption (Venkatesh et al., 2003).

While the UTAUT has been validated in various contexts and settings (Venkatesh et al., 2016), most studies have relied on student and technology-specific user populations, using generic moderators, such as age and gender (Lee et al., 2003). Research conducted in workplace settings is less extensive, although it is increasing (King and He, 2006; Chuttur, 2009; Venkatesh et al., 2016). Results also vary among settings (King and He, 2006). In general, UTAUT has been found to predict approximately 70 percent of variation in behavioral intention (Venkatesh et al., 2003) and around 50 percent in technology use (Venkatesh et al., 2016).

Alongside studies in technology adoption, research on employee perceptions and attitudes relating to technological change and digital disruption in general is growing. This is a critical factor to take into account since attitudes to discrete technologies can be shaped by overall attitudes to broader technological transformations in society and their impact on jobs. Employee attitudes to disruption have long been studied within sectors such as manufacturing and automotive engineering (Chao and Kozlowski, 1986; Haddad, 1996; Gurtoo and Tripathy, 2000) media and libraries (Jones, 1999; Karimi and Walter, 2015), which were among some of the first to undergo technological change. However, recent developments in disruptive technologies are increasingly disrupting a larger variety of sectors, including financial services (Veiga et al., 2014), health care (Blease et al., 2018), and service sectors (Di Pietro et al., 2014), among others.

## The Impact of Digital Transformation on Work-Related Outcomes

Despite important theoretical advancements in understanding technology acceptance, there has only been modest integration of this body of research and other employee-related factors likely to influence current understanding. Instead, existing digital transformation models primarily focus on the technology process and strategy (Agarwal et al., 2010; Matt et al., 2015; Berghaus and Back, 2016) and omit integration of other factors. The impact of technology on employee-and work-related outcomes has been identified as an important direction for research (Venkatesh, 2006; Venkatesh and Bala, 2008), although until recently, few frameworks have been developed or tested. Recently, Kaasinen et al. (2018) developed a worker-centric design and evaluation framework for Industry 4.0, integrating research on technology acceptance with work-related wellbeing indicators such as job satisfaction and work engagement, drawing on prior models of work-related wellbeing (e.g., Danna and Griffin, 1999). The framework proposes antecedents at the individual, organizational and environmental levels that have immediate implications for a worker’s experience with the technology or procedure (i.e., user acceptance, user experience, usability and safety). These in turn impact work-related wellbeing and organizational outcomes (Kaasinen et al., 2018). As organizations digitally transform, employers will need to pay increasing attention to employee well-being. Additional individual factors, such as workplace resilience and adaptability, are also likely to influence digital transformation outcomes for individuals and organizations alike but have not been well studied in relation to digital transformation.

Increased uptake of advanced technology is accompanied by growing skills shortages in the labor market, where reskilling and upskilling employees is one of the most critical challenges that organizations and governments face. Leading industry reports predict that most companies will have increasing skills gaps in the years to come, with employers now seeking employees with a range of skills, such as critical thinking, analytic and problem-solving skills, alongside self-management, adaptability and resilience (World Economic Forum, 2020; McKinsey, 2021). A recent survey by McKinsey (2021) found that most companies globally (89 percent) have a skills gap or will have one in the next few years. Alongside greater demand for highly specialized skills (Chuang and Graham, 2018), employers also emphasize critical thinking, analytic and problem-solving skills, self-management, adaptability and resilience as top skills needed in today’s workforce (World Economic Forum, 2020). Individuals’ abilities to acquire new skills and their receptiveness to training are thus another important priority for research attention as digital transformation increases.

### Group Dynamics and Organizational Factors Impacting Digital Transformation

Alongside the inclusion of employee-factors and work related outcomes, there is a need for multidisciplinary frameworks that integrate multiple factors across other levels, such as group dynamics and organizational level process and outcomes (Venkatesh, 2006; Chan, 2019). Such a multidisciplinary and multi-level research focus accords with broader trends in organizational behavior (Kozlowski and Klein, 2000; Klein et al., 2001; Ployhart, 2012; Johns, 2018), including the need for closer investigation of the intersections between individual, group and organizational factors in technological transformation (Seers et al., 1995; Avolio et al., 1999; Burton-Jones and Gallivan, 2007; Venkatesh et al., 2016). Overall, we need to better tease out the linkage between technology as a driving force underpinning digital transformation and its impact on workers and organizations as a whole.

Existing models of organizational behavior (OB) examine and predict human behavior in workplace settings and are useful for understanding factors that affect individuals and organizations at multiple levels. OB frameworks examine human behavior and organizations across three levels: (1) individuals in organizations (micro-level): (2) work groups (meso-level); (3) how organizations behave (macro-level) (Wagner and Hollenbeck, 2010). OB builds on contributions from a number of behavior disciplines, including psychology, which looks primarily at the individual or micro-level. Other disciplines such as social psychology, sociology and anthropology, contribute to understanding of meso and macro concepts such as group and organizational processes and outcomes (Robbins and Judge, 2019). Topics studied within organizational behavior commonly include employee attitudes and engagement, identification and commitment, motivation, culture and climate, leadership, group and teams relationships, and health and well-being, among others (Ployhart, 2015). Additionally, scholars have recently highlighted the importance of human capital to existing OB models. Human capital exists at the individual level, in terms of expertise, skills and competencies, but also spans other organizational levels, such as resources and support for training and talent development (Ployhart, 2015). Given rising concerns about skills gaps in the context of 4IR and the future of work, much can be learned from integrating current frameworks for Industry 4.0 (e.g., Kaasinen et al., 2018; Molino et al., 2020) with existing models of organizational behavior (Robbins and Judge, 2019).

## Review Aims and Methods

In this paper, our aim is provide fresh theoretical understanding (Webster and Watson, 2002) of digital transformation as a topic that has received considerable attention in practice, yet lacks conceptual clarity, particularly as it relates to workplace factors rather than business or strategic processes. By reviewing literature across multiple disciplines and examining factors that may support or inhibit digital transformation across different organizational levels, we seek to extend IS and business-focused research on digital transformation by further incorporating insights from psychology, organizational behavior, and management studies. Our goal is to consolidate and synthesize current theory and empirical research into an overarching, multi-level theoretical framework for digital transformation. In turn, we aim to guide further research, practice and policy on digital transformation as a new and urgent imperative facing organizations and society as a whole.

We theorize that digital transformation is influenced by multiple factors at the individual, group and organizational level. Drawing on models of organizational behavior and management (e.g., Robbins and Judge, 2019). Through preliminary scoping of academic and gray literature (i.e., industry trends), we considered five overarching factors related to effective digital transformation at the individual level. These are technology acceptance; perception and attitudes toward technology and digital transformation; skills and training; workplace resilience and adaptability, and work-related wellbeing. At the work group-level, we theorized that effective digital transformation is supported by three main factors: team communication and collaboration; workplace relationships and team identification, and team adaptability and resilience. At the organizational level, we theorized that three overarching factors in supporting an organization’s digital transformation: leadership; human resources; organizational climate, and culture.

We then conducted a targeted search of each factor, reviewing theory as well as empirical studies related to digitalization or digital transformation in workplace settings. Specifically, due to this review’s broad scope and the multidisciplinary and multi-level nature of digital transformation, we have attempted to balance both the depth and breadth of existing theory and research. We conducted a title, abstract, and keyword search of the ScienceDirect database using synonyms for 1) digital, 2) workplace, and 3) transformation. To ensure that we review recent literature, we limited our search to English publications from 2000 to August 2020. Additionally, we manually searched reference lists of reviews on digital transformation and relevant highly cited publications, and conducted “ancestry and snowballing” citation tracking (Greenhalgh et al., 2005, p. 5; Greenhalgh et al., 2017). This search strategy ensured that we searched on digital transformation more generally to understand research trends and were able to identify studies focusing on individual factors. We did not aim to be exhaustive but rather strove to highlight current research and trends to inform future research and theory development. Thus, we only included empirical studies published in high-quality journals (i.e., impact factor greater than 1) and after assessing the study’s methodological rigor. We limited our pool to studies focusing on workplaces as the primary research setting and that investigated individual, group, or organizational level factors relevant to digitalizing workplaces. We excluded studies reporting on non-workplace or worker contexts and studies of digital or physical workplace design interventions (e.g., ergonomics, digital wellbeing interventions).

## Review Findings

We organized our findings into three levels: individual, group, or organizational level. The findings are summarized in Table 1 according to each factor and we present workplace studies conducted after 2000, with review studies shown with an asterix. Following presentation of findings, we organize the three factors into a multi-level framework, showing linkages between the three levels and possible moderating factors.

**TABLE 1:** Summary of identified articles, with* indicating a review article.

**Factor**	**Identified articles**
**Individual level**	
Technology adoption	
Attitudes and perceptions relating to technological change	Blease et al. (2018); Bond et al. (2018); Brougham and Haar (2017, 2018); Cascio and Montealegre (2016)*; Cadwallader et al. (2010); Di Pietro et al. (2014); Doraiswamy et al. (2018); Hettich (2017); Li et al. (2018); Li et al. (2019); Mercader and Gairín (2020); Meske and Junglas (2020); Niedzwiecka and Pan (2017); Sarwar et al. (2019); Schraeder et al. (2006); Tasdogan (2020); Vieitez et al. (2001)
Skills and training	Bakker et al. (2012); Beer and Mulder (2020)*; Berg and Chyung (2008); Blume et al. (2010)*; Bolívar-Ramos et al. (2012); Bode and Gold (2018); Börner et al. (2018); Brown and Souto-Otero (2020); Brunetti et al. (2020); Cascio (2019)*; Chauhan et al. (2016); Ederer et al. (2015); Gamrat et al. (2014); Gorlitz and Tamm (2016); Grundke et al. (2018); Harteis and Goller (2014); Li and Herd (2017); Martín-Rojas et al. (2019); Melián-González and Bulchand-Gidumal (2017); Mercader and Gairín (2020); Noe et al. (2014)*; Oberlander et al. (2020)*; Osmundsen (2020); Sousa and Rocha (2019)*
Workplace resilience and adaptability	Baard et al. (2014)*; Badran and Youssef-Morgan (2015); Burns et al. (2013); Britt et al. (2016)*; Cameron and Brownie (2010); Cullen et al. (2014); Ferris et al. (2005); Fisher et al. (2018)*; Förster and Duchek (2017); Guo et al. (2017); Harms et al. (2017); Hartmann et al. (2020)*; Huang et al. (2014)*; Jensen et al. (2008); Jung and Yoon (2015); Kinman and Grant (2011); Kossek and Perrigino (2016); Lamb and Cogan (2016); Larson and Luthans (2006); Lounsbury et al. (2003); Luthans et al. (2005); Luthans et al. (2007); Luthar et al. (2000); Lyons et al. (2015); Mache et al. (2014); Malik and Garg (2017); McDonald et al. (2016); Ployhart and Bliese (2006); Seligman and Csikszentmihalyi, 2000; Stevenson et al. (2011); Wanberg and Banas (2000); Wei and Taormina (2014); Welbourne et al. (2015); Yang and Danes (2015); Youssef and Luthans (2007).
Work-related stress and wellbeing	Ayyagari et al. (2011); Bakker and Demerouti (2007)*; Bakker and Demerouti (2017)*; Bouckenooghe et al. (2013); Bowling et al. (2010)*; Diener et al. (2018)*; Edmans (2012); Field and Chan (2018); Fisher (2003); Jena (2015); Kazmi et al. (2008); Koys (2001); Harter et al. (2002)*; Judge et al. (2001)*; Kagan (2016); Kinicki et al. (2002)*; Krekel et al. (2019)*; Lepine et al. (2005)*; Liu et al. (2019); Nisafani et al. (2020)*; Ragu-Nathan et al. (2008); Riketta (2008)*;Schneider et al. (2003); Silvestro (2002); Tarafdar et al. (2010); Tarafdar et al. (2019)*; Tarafdar et al. (2015); Tenney et al. (2015); Tenney et al. (2016)*; Wright et al. (2002); Wright et al. (2007); Zeike et al. (2019a); Zeike et al. (2019b)
**Group**	
Team communication and collaboration	Alshawi and Ingirige (2003)*; Anders (2016); Banker et al. (2006); Berghaus and Back (2016); Bolstad and Endsley (2003)*; Boughzala et al., 2012; Boughzala and de Vreede (2015); Ellison et al. (2014); Faems et al. (2005); Fletcher and Major (2006); Gibson (2001); Grudin (2006)*; Guinan et al. (2019); Hur et al. (2019); Jordan et al. (2002); Kirkman and Mathieu (2005)*; Kozlowski and Ilgen (2006)*; Leonardi et al. (2013)*; Lloréns-Montes et al. (2005); Marlow et al. (2018)*; Merschbrock and Munkvold (2015); Mesmer-Magnus and DeChurch (2009)*; Nam et al. (2009)
Workplace relationships and team identification	Agarwal Upasna et al. (2012); Atitumpong and Badir (2018); Cole et al. (2002); Fay and Kline (2011); Hartmann et al. (2020)*; Huang and Liu (2017); Janssen and Huang (2008); Liao et al. (2010); Leonardi et al. (2013)*; Mukherji and Arora (2017); Sanders et al. (2010); Schlagwein and Hu (2017); Sias and Duncan (2018); Sias (2009); Sias and Perry (2004); Treem and Leonardi (2012)*; Tripsas (2009); Tyworth (2014); Utesheva et al. (2016); van Der Vegt and Bunderson (2005); Yanez Morales et al. (2020)
Resilience and adaptability	Carmeli et al. (2013); Hartmann et al. (2020)*; Marks et al. (2001); Meneghel et al. (2016a); Meneghel et al. (2016b); Stephens et al. (2013); Stoverink et al. (2018)
**Organizational-level**	
Leadership	Bartol and Liu (2002); Bass et al. (2003); Berson and Avolio (2004); Carreiro and Oliveira (2019); Chanias et al. (2019); Cortellazzo et al. (2019)*; Dery et al. (2017); Elenkov et al. (2005); Gemeda and Lee (2020); Haddud and McAllen (2018); Hess et al. (2016); Matt et al. (2015); Roepke et al. (2000); Yukl (2006); Zaccaro and Klimoski (2002)
Human Resources	Benson et al. (2002); Chuang and Graham (2018)*; Grant and Newell (2013); Hess et al. (2016); Li and Herd (2017); Marler and Fisher (2013)*; Marler and Boudreau, 2017*; Noe et al. (2014)*
Organizational culture and climate	Beus et al. (2020)*; Büschgens et al. (2013)*; Brunetti et al. (2020); Chanias et al. (2019); Denison et al. (2014)*; Dery et al. (2017); Hartnell et al. (2011)*; Hartl and Hess (2017); Jung et al. (2003); Mueller and Renken (2017); Osmundsen et al. (2018)*; Ostroff et al. (2003)*; Patterson et al. (2005); Schein (2004); Schneider et al. (2013)*; Zohar and Hofmann (2012)*

### Individual-Level

At the individual level, we propose five overarching factors related to effective digital transformation among employees: technology adoption; perception and attitudes toward technology and digital transformation; skills and training; workplace resilience and adaptability, and work-related wellbeing.

#### Technology Acceptance and Adoption

In the workplace, technology acceptance and adoption has been studied in a range of settings, including manufacturing and construction (Venkatesh and Davis, 2000; Son et al., 2012), hotels (Lam et al., 2007; Hong et al., 2011), banking and financial services (Liao and Landry, 2000; Brown et al., 2002; Veiga et al., 2014), higher education (Talukder, 2012), IT services/consulting (Kim and Kankanhalli, 2009), government (Burton-Jones and Hubona, 2006), postal services (Dutta and Borah, 2018), and real estate (Venkatesh, 2000). Several studies also explored technology adoption across multiple settings (e.g., Venkatesh and Davis, 2000; Venkatesh et al., 2003; Burton-Jones and Hubona, 2006; Kim et al., 2007; Jones et al., 2010; Lee et al., 2013; Wang et al., 2014). The technology studied in workplaces includes general information technology (IT) (Liao and Landry, 2000; Kim et al., 2007, 2017; Lam et al., 2007; Dutta and Borah, 2018) or specific technologies, such as email and word processing software (Venkatesh and Davis, 2000; Burton-Jones and Hubona, 2006) and IS systems such as agile IS and e-learning (Hong et al., 2011; Lee et al., 2013).

In workplace settings, studies of technology adoption have found that the nature of technology matters, such as whether technology use is voluntary or mandatory (Lee et al., 2003; Chuttur, 2009). Venkatesh and Davis (2000) seminal work explains that subjective norms are more salient in mandatory systems. In voluntary settings, perceptions of the technology and subjective norms will influence adoption intentions and resultant technology use. However, in mandatory settings, technology adoption occurs regardless, but these perceptions will affect attitudes toward technology and may be more profound, with broader organizational impacts (Brown et al., 2002). Specifically, when employees perceive that the technology will be useful to their work and help them to perform, and is easy for them to learn and use, the odds of adoption increase (Burton-Jones and Hubona, 2006; Wang et al., 2014). Consistent with studies conducted in other settings on the perceived usefulness of technology and its ease-of use, notably, there is an established link between user satisfaction and IT adoption in the workplace too (Liao and Landry, 2000; Kim et al., 2007; Son et al., 2012). These findings imply that new technology and systems should ideally be useful and easy for employees to use, whether mandatory or not.

One way to resolve questions of perceived usefulness versus ease of use of technology in workplace settings is to consider how employees might experience technology adoption differently. Dutta and Borah (2018) found that IT adoption varied by gender, age and experience. In particular, male employees were more comfortable operating IT at work, while female employees were more encouraging of IT changes, especially those with longer work experience. Employees who had served longer in the organization (more than 30 years) were more anxious about working with IT but generally accepted IT due to peer and social pressure. Interestingly, older employees with longer work experience (i.e., about 20–30 years) were highly satisfied with IT usage.

Another aspect to focus on is the fit between the technology and tasks employees perform as this fit influences employees’ attitudes and technology adoption (Lam et al., 2007). In a longitudinal study of the adoption of a new enterprise system software, Veiga et al. (2014) found that employees who expected the system to help them perform better at work and open the door to job opportunities or job security were more likely to use it and continue to enhance their knowledge post-adoption. In addition, the perception of organizational support for the system had polarizing effects on adoption, increasing the positive perception of the system among adopters but decreasing the usage among non-adopters. In other words, organizations must exercise care in introducing new technologies so that they win the support of adopters but without alienating the non-adopters. In a study of blue collar workers, Molino et al. (2020) found that both personal resources, such as resilience, along with organizational resource, such as opportunities for information and training, led to greater technology acceptance. The results demonstrate the value of providing all employees with knowledge and training opportunities to facilitate digital transformation without affecting the motivation of workers (Molino et al., 2020).

In the context of digital transformation today, new technologies are introduced in increasingly shorter cycles and often concurrently. This requires a different perspective on technology adoption. Notable drivers of acceptance of agile IS include an individual’s level of comfort with constant changes, their innovativeness, as well as other facilitating conditions afforded by the technology and workplace (such as maintaining consistency between systems and having management support) (Jones et al., 2010; Hong et al., 2011).

Increasingly, new technologies introduced in workplaces have collaborative and social networking functions (e.g., virtual discussion rooms, forums, and chat functions) whose successful adoption is contingent on employees adopting them collectively. Talukder (2012) showed that peer social networks, including fellow employees and management, can influence attitudes toward an innovation and, ultimately, its adoption. These studies highlight the growing importance and the challenge of creating positive social norms around technology use to facilitate technology adoption.

#### Perceptions and Attitudes Toward Technological Change

Alongside studies in technology adoption, research on employee perceptions and attitudes relating to technological change and digital disruption in general is growing. This is a critical factor to take into account since attitudes to discrete technologies can be shaped by overall attitudes to broader technological transformations in society and their impact on jobs. Employee perceptions and attitudes toward technological change continues to be studied within the manufacturing and automotive sectors which were among the first to automate (Vieitez et al., 2001; Kim and Kim, 2018). However, recent developments in new technologies such as AI, robotics, and cloud computing are increasingly disrupting a large variety of sectors, including health care (Blease et al., 2018; Doraiswamy et al., 2018; Sarwar et al., 2019; Tasdogan, 2020), wholesale and service sectors (Hettich, 2017; Li et al., 2018; Meske and Junglas, 2020), banking/financial services and education (Niedzwiecka and Pan, 2017; Bond et al., 2018; Mercader and Gairín, 2020).

In general, studies have shown that higher perceptions of job insecurity due to new technologies are negatively associated with organizational commitment and career satisfaction and positively associated with cynicism, depression, and turnover intentions (Vieitez et al., 2001; Brougham and Haar, 2018; Li et al., 2019). However, these findings differ across organizational settings, job roles, and other contextual factors, such as gender, age, and technology type. Importantly, studies have shown that employees who were engaged in making decisions related to the technology changes reacted more positively to the changes than individuals with lower levels of involvement (Schraeder et al., 2006).

In healthcare settings, recent surveys have found medical physicians to be both skeptical and optimistic about new digital technologies, such as AI (Blease et al., 2018; Doraiswamy et al., 2018; Sarwar et al., 2019; Tasdogan, 2020). On the whole, physicians were not overly concerned about their jobs becoming obsolete and were doubtful about the potential of technology to outperform humans and replace human clinicians in delivering care (Blease et al., 2018; Doraiswamy et al., 2018; Tasdogan, 2020). However, physicians did believe that new technologies would change their professions (Sarwar et al., 2019; Tasdogan, 2020) and were optimistic about technology’s potential as a diagnostic tool (Sarwar et al., 2019) and to improve healthcare delivery and relieve administrative burdens (Blease et al., 2018). Some respondents thought documenting and updating medical records could be replaced by AI and machine learning technologies (Doraiswamy et al., 2018). In two multi-country studies, findings varied according to gender and country location. In one study, female and US-based doctors were more pessimistic about technology risks outweighing benefits (Doraiswamy et al., 2018), while in another, males and more experienced practitioners were more optimistic about the integration and adoption of AI into practice (Sarwar et al., 2019).

In the service sector, there is evidence that employees are generally motivated to support new technologies such as self-service technologies (Cadwallader et al., 2010; Di Pietro et al., 2014; Hettich, 2017). In a qualitative study, Di Pietro et al. (2014) found that employees evaluated that self-service technologies improved their productivity at work while also increasing their scope of work (e.g., hours, increased sales/clients and client satisfaction) and enhanced the quality of work (more satisfying, enhanced and faster transactions). However, Hettich (2017) found that attitudes toward self-service technologies are moderated by job type and nature of automation (e.g., automating routine tasks). Technology that is perceived as leading to future job loss or reductions is more likely to elicit negative attitudes (Brougham and Haar, 2017, 2018; Hettich, 2017).

In other studies, the mere awareness of new technologies (e.g., smart technology, AI, automation, robotics, and algorithms) by employees was generally related to perceptions of potential job redundancy, increased turnover intentions, cynicism and depression, and lower levels of organizational commitment and career satisfaction (Brougham and Haar, 2017, 2018; Hettich, 2017; Li et al., 2019). For example, Li et al. (2019) found that AI and robotics awareness were significantly associated with employee turnover intention. However, this relationship was moderated by perceived organizational support and competitive psychological climate (Li et al., 2019).

Recent reviews (Cascio and Montealegre, 2016) have highlighted the importance of job role and work-design factors in digitalizing workplaces. Vieitez et al. (2001) found a relationship between perceptions of job security and wellbeing in the process of technological change. However, perceived threats to job security were influenced by personal and situational characteristics such as formal training, type of work department, professional categories and the type of technology used. Research on attitudes toward digital transformation is more scarce. However, in a study of work design characteristics, Meske and Junglas (2020) found that employees’ expectations of autonomy, competence, and connectedness in the digital workplace were linked to increased support for digital transformation.

#### Skills and Training

Advancements in new technologies are shifting the types of skills and competencies needed in the workplace. Individuals’ abilities to acquire new skills and their receptiveness to training are thus another focus of research attention. Digital competencies are defined as a set of basic knowledge, skills, and abilities that allow workers to perform and complete their job tasks within digital work environments (Oberlander et al., 2020). Along with commonly used technologies such as document processing and email, employees are now required to use a wider range of software packages and digital tools (Harteis and Goller, 2014; Brown and Souto-Otero, 2020; Brunetti et al., 2020). Meanwhile, as more organizations undergo digital transformation, the need for highly specialized technical skills in areas such as software development, AI and data analytics, nanotechnology, robotization, IoT, and cybersecurity is increasing (Sousa and Rocha, 2019). A survey of LinkedIn professionals also found that technical skills in AI, nanotechnology, robotization, and IoT, and being proficient in digital learning contexts such as mobile technologies, tablets, and smartphones are more important among employers (Sousa and Rocha, 2019).

Alongside these trends, there is growing emphasis on the importance of soft skills such as communication, problem-solving, and creativity in technology-rich environments (Ederer et al., 2015; Börner et al., 2018; Grundke et al., 2018). Notably, Osmundsen (2020) found that cognitive competencies such as a willingness to learn and openness to change were critical in digital competencies as a prerequisite for digital capabilities in areas such as robotization, machine learning, sensor technology, and big data.

This growing emphasis on soft skills could explain the apparent mismatch between education and training and the types of skills now required in the workplace (Börner et al., 2018; Brown and Souto-Otero, 2020). For example, recent big data analyses of job advertisements and course syllabi have found that social skills, specific technical skills, and personality traits, rather than academic qualifications, are increasingly in demand (Brown and Souto-Otero, 2020). Similarly, Börner et al. (2018) found that soft-skills such as problem-solving, organizational skills, customer service, and writing feature more prominently in job ads (Sousa and Rocha, 2019). A recent systematic review by Beer and Mulder (2020) also found that information processing enabled by technology has created increasing demands for cognitive skills (e.g., synthesizing and interpreting data) and interpersonal skills (e.g., coordinating and monitoring other people). However, the demand for manual, psychomotor skills (e.g., manual producing and precise assembling) is decreasing. Moreover, the standardization of work is positively related to interpersonal skills, but not related to cognitive and psychomotor skills, while higher task variety is positively related to the demand for cognitive and interpersonal skills, rather than psychomotor skills (Beer and Mulder, 2020).

The willingness to learn new skills is therefore a positive trait that employers seek. At the individual level, learning can be formal or informal, planned or spontaneous, and conscious or unconscious, with recent studies finding that learning is becoming more continuous, informal, and self-directed (Noe et al., 2014; Sousa and Rocha, 2019). Informal learning is defined as a cognitive activity or behavior, such as learning through self-reflection or from others, including peers, supervisors, and mentors (Noe et al., 2014). Berg and Chyung (2008) found that employees’ interest in their professional field, rather than monetary rewards for good performance has more impact on informal learning engagement. Engagement in informal learning did not vary by gender or level of education but older employees showed higher levels of engagement (Berg and Chyung, 2008). In digital contexts, workplace learning has broadened from traditional in-person training to a range of online and e-learning contexts such as websites, LinkedIn, Facebook, blended learning, and massive open online courses (MOOC), among other formats (Sousa and Rocha, 2019).

Factors such as attitudes and personality also play a role in workplace learning and training transfer, defined as the extent to which the learning from training transfers to job outcomes, such as changes in work performance (Blume et al., 2010; Ford et al., 2018). A meta-review by Blume et al. (2010) found that training transfer is positively related to cognitive ability, conscientiousness, motivation, and a supportive work environment, while factors such as motivation and work environment had a stronger relationship to transfer based on the focus of training (e.g., leadership development versus computer software training).

Other reviews have found that conditions such as whether training is voluntary, co-workers’ attitudes, and whether workers have input on training design and post-training opportunities impact workers’ motivation and learning, such as efforts to positively transfer newly learned skills to the job (Cascio, 2019). Other studies have found that work engagement is positively related to task performance and active learning, particularly for employees high in conscientiousness (Bakker et al., 2012). Employees might also benefit from personalized learning and training within increasingly digitalized environments. The advancement of digital technology has also led to changes in workplace learning environments, such as the increasing use of platform-based technologies that allow learners “to personalize their learning space” and gain increased access to learning opportunities (Li and Herd, 2017, p. 186). For example, studies have found that personalized professional development within the education sector, such as digital badging, supported teachers in selecting their own learning goals and customizing learning activities and training (Gamrat et al., 2014). Other research within higher education found that rather than personality traits, lack of training in digital competencies (e.g., time management, training, pedagogical approaches, experience, and teaching approaches) in using digital technologies was more salient (Mercader and Gairín, 2020).

Concerning types of job and job tasks, there is evidence that adaptive and self-directed learning is more common in highly skilled workers, who are also more likely to participate in training (Gorlitz and Tamm, 2016). For example, Gorlitz and Tamm (2016) found that workers with non-routine tasks (e.g., nursing, service and healing, training, educating, planning, and negotiating) were more likely to participate in training than those doing routine tasks (e.g., fabricating and producing goods, supervising and controlling machines, repairing and patching). Harteis and Goller (2014) found that employment type (i.e., more highly skilled workers) received more support for workplace learning, regardless of age or gender. Worker personality traits such as openness to new experience and emotional stability were also found to be less susceptible to the effects of digitalization (Bode and Gold, 2018). These findings suggest that less skilled workers need more encouragement and support to upskill.

At the group level, results confirmed the importance of supervisor support in the transfer of skills and training; however, peer support was greater than that of supervisors (Chauhan et al., 2016). In firm studies, support from top management and technological skills and competencies were linked to organizational learning, corporate entrepreneurship, and firm performance (Bolívar-Ramos et al., 2012; Martín-Rojas et al., 2019). In service industries, front-office workers are increasingly using technology in their roles but because human participation is still necessary employees need training in the adoption of technologies, alongside training in non-routine and face-to-face tasks and interactions (Melián-González and Bulchand-Gidumal, 2017).

#### Workplace Resilience and Adaptability

Resilience is the dynamic process of adapting and coping during significant adversity (Luthar et al., 2000; Harms et al., 2017) and builds on the tenets of positive psychology (Seligman and Csikszentmihalyi, 2000). Although individual resilience is both a personality trait and a capacity that can be developed, recent evidence suggests that resilience might be better conceptualized as a developmental process (Hartmann et al., 2020). This is because resilience may present differently across various work-life domains (Harms et al., 2017) and is influenced by resilience mechanisms (e.g., coping strategies) and resilience promoting factors (e.g., personal and environmental characteristics) (Kossek and Perrigino, 2016; Fisher et al., 2018; Hartmann et al., 2020). The potential for resilience to be cultivated can allow an individual to overcome adversity to perform as well as before, if not better, and regain “a steady state of wellbeing” (Britt et al., 2016; Hartmann et al., 2020, p. 6; Luthar et al., 2000).

In the workplace, four categories of antecedents influence individual resilience (Hartmann et al., 2020). First, certain personality traits (e.g., future-orientation, conscientiousness, openness to experience, and emotional stability (Wei and Taormina, 2014; Lyons et al., 2015) and cultural values (Wei and Taormina, 2014; Welbourne et al., 2015) are positively linked to resilience. Second, personal attributes such as self-efficacy and possessing an internal locus of control (Lyons et al., 2015; Guo et al., 2017), confidence in being able to address challenges at work (Yang and Danes, 2015), the ability to manage work demands, establish work-life balance and be reflective (Jensen et al., 2008; Kinman and Grant, 2011) are related to resilience. Third, an individual’s attitude and mindset toward their job and professional development help them become resilient during adversity (Cameron and Brownie, 2010; Stevenson et al., 2011). Lastly, the work context (e.g., the presence of social support, feedback, sharing of responsibilities and work tasks (Cameron and Brownie, 2010; Burns et al., 2013; Lamb and Cogan, 2016; McDonald et al., 2016; Förster and Duchek, 2017) are related to resilience among employees.

Individual resilience is important because it is related to job performance (Luthans et al., 2005, 2007), organizational citizenship behavior (Jung and Yoon, 2015), organizational commitment (Larson and Luthans, 2006; Youssef and Luthans, 2007), work engagement (Mache et al., 2014) and openness and commitment to organizational change (Wanberg and Banas, 2000; Malik and Garg, 2017). As discussed further below, these factors are likely to contribute toward more successful digital transformation. Finally, cultivating resilience supports employee retention and is positively related to job and career satisfaction (Lounsbury et al., 2003; Youssef and Luthans, 2007; Badran and Youssef-Morgan, 2015; Lyons et al., 2015) and promotes positive mental health (Kinman and Grant, 2011) and physical well-being (Ferris et al., 2005).

Adaptability at work is a related concept to resilience and can be viewed either as the performance by a worker (i.e., the ability to adapt and perform) or as a characteristic of the individual (i.e., a determinant of work performance) (Ployhart and Bliese, 2006). However, both are important as the nature of work changes (Charbonnier-Voirin et al., 2010). At the individual level, adaptive performance includes being able to make cognitive, affective, motivational, and behavioral adaptations when tasks or work demands change (Baard et al., 2014). Individual adaptability helps workers perform better at work because adaptable workers are more proactive and take responsibility for adjusting to changing situations and are more likely to positively perceive these situations (Ployhart and Bliese, 2006; Cullen et al., 2014). There are known factors that contribute to individual adaptability. For instance, the personality traits of openness to experience, emotional stability, conscientiousness, and ambition are positively related to individuals’ adaptive performance (Pulakos et al., 2006; Huang et al., 2014).

#### Work-Related Stress and Wellbeing

In the face of technological change and digital transformation, it is essential to consider the adverse impacts of technology on work-related stress and wellbeing since these will have bearing on employee performance and job satisfaction. In general, stress is often found to be associated with lower levels of performance (Kazmi et al., 2008). However, it depends on where the stress originates. Stress arising from good challenges (e.g., taking on a new project) is less detrimental than stress due to bureaucracy or role ambiguity (Lepine et al., 2005). Acute episodes of stress and their relationship with performance are potentially an inverted U-shape (Kazmi et al., 2008; Kagan, 2016). While the relationship between stress and performance is complex, it is clear that stress and poor mental health are related to lower levels of work performance (Tenney et al., 2016).

When implementing digital technologies, stress can result in a phenomenon called technostress, defined as stress that individuals experience due to their use of technology and the inability to cope or deal with these new digital technologies in a healthy manner (Tarafdar et al., 2015; Nisafani et al., 2020). The causes of technostress include dependency on technology when working (Liu et al., 2019), work overload (Tarafdar et al., 2010), anxiety about one’s own IT capabilities amidst constantly changing technology (Ragu-Nathan et al., 2008) and work-home conflict (Ayyagari et al., 2011). For example, the adoption of digital technology has led to the fragmentation of work and produced a perpetual sense of urgency and increased blurring of work-life boundaries (Field and Chan, 2018). Similarly, the rise of email, smartphones, and new messaging software such as WhatsApp has increased communication and collaboration while creating expectations that employees need to always be available, including outside of office hours (Ayyagari et al., 2011). Consequently, workers experiencing technostress report lower productivity, wellbeing, and commitment to the organization (Jena, 2015; Nisafani et al., 2020). However, recently it has been suggested that technostress could also lead to positive outcomes at work, improving effectiveness and fostering innovation (Tarafdar et al., 2019), as digital technologies - when designed appropriately - can also mitigate technostress and create positive effects on workers (Tarafdar et al., 2019).

Subjective wellbeing is commonly referred to as happiness or satisfaction and is constituted by people’s appraisals and evaluation of their own lives (Diener et al., 2018). It has been shown to be related to work performance (Judge et al., 2001; Kinicki et al., 2002; Riketta, 2008) and can be examined across life as a whole or in specific facets including at work (e.g., job satisfaction, positive affect at work, and absence of job stress or negative affect at work (Bowling et al., 2010). Employees with higher job satisfaction perform better at work than their unhappy colleagues (Fisher, 2003; Wright et al., 2007; Bouckenooghe et al., 2013; Tenney et al., 2015, 2016). Higher subjective wellbeing may also lead to optimism and self-efficacy, which increases task persistence and enhances learning, leading to better performance over time, resilience and adaptability as digital transformation takes place (Tenney et al., 2015).

Research on work design, such as the Job Demands-Resources (JD-R) model (Bakker and Demerouti, 2007, 2017) may be particularly useful in understanding the impact of technology and digital transformation on work-related wellbeing. According to JD-R theory, two key factors influence work environments: job demands, which include physical, psychological, social, or organizational aspects of a job that require sustained effort or skills; and job resources, defined as aspects of the job that support work goals, reduce job demands, and stimulate learning and development (Bakker and Demerouti, 2007). Excessive job demands, when not accompanied by adequate resources, can lead to reduced health and a higher risk of burnout and lower levels of work engagement and wellbeing. In a study of leaders, Zeike et al. (2019b) found an association between perceived choice overload (e.g., the burden of leadership decisions and complexity of choice), pressure from digitalization (e.g., pressures to keep up with the latest technologies and prepare for digitization) and psychological wellbeing. However, in another study, leaders who were better skilled in digital leadership had higher levels of wellbeing, regardless of gender, age, and managerial experience (Zeike et al., 2019b).

There is comparatively less research exploring the relationship between subjective wellbeing and performance at the organizational level. However, the limited evidence available suggests that employee subjective wellbeing predicts an organization’s performance. There is a positive relationship between employee subjective wellbeing and aggregate, organizational-level measures of performance (Koys, 2001; Schneider et al., 2003; Edmans, 2012), customer satisfaction, productivity, and absenteeism (Harter et al., 2002; Krekel et al., 2019). This relationship has been observed across different industries and is particularly strong in customer satisfaction and staff turnover, both of which drive overall profitability (Krekel et al., 2019). However, other studies have failed to find a relationship between subjective wellbeing and individual or organizational performance (Silvestro, 2002; Wright et al., 2002), which is more likely due to the effects of moderators in this relationship. Tenney et al. (2016) proposed the presence of at least seven moderators on wellbeing and organizational performance: the health of the individual, absenteeism, the ability to self-regulate, motivation, creativity, personal and social relationships, and turnovers.

### Group-Level

Next, we go beyond the individual employee to consider work groups. At the group-level, we present three factors to support effective digital transformation: team communication and collaboration; workplace relationships and team identification, and team adaptability and resilience.

#### Team Communication and Collaboration

Team collaboration refers to the joint effort of a group of people toward common goals, whereby two or more agents share resources, skills, discoveries and are responsible for the shared outcome (Briggs et al., 2003; Boughzala et al., 2012). In the workplace, the quality of team collaboration can be assessed according to five key dimensions, namely people, process, leadership/management, information, and technology (Boughzala et al., 2012). Previous studies have revealed that team collaboration constitutes one of the essential elements of organizational functioning. The quality of collaborative work practices relates to organizational performance and productivity (Jordan et al., 2002; Banker et al., 2006; Boughzala and de Vreede, 2015). Effective collaboration among co-workers is also found to positively link to high levels of innovative performance in work teams and organizations (Faems et al., 2005; Lloréns-Montes et al., 2005).

Communication among team members is a crucial element of successful team collaboration. In the existing literature, team communication is usually measured by quality and frequency (Marlow et al., 2018). Effective team communication facilitates intra-team information flow, idea exchange and task integration and thereby serves as a support mechanism for many other team processes such as task coordination, collective decision making, clarifying misunderstandings, and so forth (Gibson, 2001; Fletcher and Major, 2006; Kozlowski and Ilgen, 2006). Team communication is also categorized as task-oriented communication, which focuses on completing tasks, achieving common goals, and relational communication, emphasizing building interpersonal relationships among team members (Nam et al., 2009; Marlow et al., 2018).

Team communication and collaboration can occur in both face-to-face encounters and mediated interactions via electronic tools (Bolstad and Endsley, 2003; Mesmer-Magnus and DeChurch, 2009). Kirkman and Mathieu (2005) propose the concept of team virtuality to capture the extent to which team members use technological tools to coordinate work tasks and the amount of informational value obtained by using such tools.

As organizations undergo digital transformation, the level of team virtuality is enhanced by implementing a variety of advanced and innovative collaboration technologies, such as video conferencing software (e.g., Skype), instant messaging (IM) platforms (e.g., WhatsApp), project management software (e.g., Slack), enterprise social media (ESM), in both geographically proximate and distributed work teams (Leonardi et al., 2013; Ellison et al., 2014; Anders, 2016). The enhanced virtuality of work team serves to facilitate both task-oriented and relational communication among team members, which in turn, engenders positive outcomes of collaborative work practices, such as efficient knowledge sharing and information flow, swift and precise task coordination, as well as increased transparency of work processes (Alshawi and Ingirige, 2003; Grudin, 2006; Ellison et al., 2014; Anders, 2016).

Based on findings from a multilevel study, Guinan et al. (2019) stressed the significance of flexible and multidisciplinary team collaboration in supporting digital transformation goals. Specifically, cross-functional and innovative ninja teams, which enable professionals from different backgrounds to collaborate in an *ad hoc* manner and deliver digital support across multiple projects, were identified as crucial digital transformation levers. Organizations also established digital hubs to accommodate teams of top-level experts in digital technology and methods to support the continuous sharing of new ideas and facilitate collaboration on digital solutions within teams. In a similar vein, Merschbrock and Munkvold (2015) revealed that the diffusion of an innovative system required the transition to a collaborative work environment characterized by clear guidelines for information exchange, appropriate allocation of roles and responsibilities, as well as stable locations and routines for cross-disciplinary exchange. Other studies have emphasized the importance of enabling employee affinity in using digital tools to collaborate and to appoint internal digital experts (Berghaus and Back, 2016). On the contrary, digital transformation processes and outcomes are likely to be impeded by obsolete team collaboration and communication habits. In particular, inertia about the pre-existing on-site collaboration and face-to-face communication routines often results in the inadaptability or even resistance to the transformation toward digitalized work and communication processes (Alshawi and Ingirige, 2003; Hur et al., 2019). Enhancing team communication and collaboration through social and technological scaffolds is therefore vital in the face of digital transformation.

#### Workplace Relationships, Team Identification

Workplace relationships refer to relationships between coworkers, including vertical supervisor-subordinate relationships and peer relationships (Sias and Perry, 2004; Sias, 2009). Supervisor-subordinate relationships are referred to as leader-member exchange (LMX) and encapsulate the reciprocal interactions characterized by mutual trust, respect, and support between a supervisor and his or her subordinates (Liao et al., 2010). Relationship between peer team members is conceptualized as team-member exchange (TMX), where teams engage in an ongoing and reciprocal exchange of ideas, feedback, and emotional support (Cole et al., 2002).

In an organization’s efforts toward successful digital transformation, the quality and style of workplace relationships can either propel or impede transformation progress. In particular, high-quality LMX can have positive effects on workplace innovations in terms by encouraging innovative work behaviors of employees (e.g., Sanders et al., 2010; Aarons and Sommerfeld, 2012; Agarwal Upasna et al., 2012; Atitumpong and Badir, 2018). TMX can also predict team members’ innovative work behaviors and performances, with the relationships mediated by various factors such as help-seeking behaviors and psychological empowerment (Yanez Morales et al., 2020). Team identification, which emerges when an individual confirms membership of a work team, is closely related to workplace relationship since employees who form close coworker relationships tend to have a stronger sense of belonging and develop identification with their work teams (van Der Vegt and Bunderson, 2005; Janssen and Huang, 2008; Fay and Kline, 2011). According to previous studies, team identification is an essential factor during the implementation of new workplace technologies. Specifically, a misalignment between features of new technology and established collective identity often results in difficulties and resistance to technology implementation, while technologies that reinforce existing identification are inclined to be well accepted and adopted by employees and the organization as a whole (Tripsas, 2009; Tyworth, 2014; Utesheva et al., 2016).

Meanwhile, the digital transformation of an organization can also affect workplace relationships and identification. In particular, the emergence of multi-functional management and communication technologies, such as ESM and IM, provides unprecedented opportunities for social engagement and value diffusion, which serve to solidify fellowship among coworkers and enhance employees’ affective attachment to their work team (Leonardi et al., 2013; Huang and Liu, 2017; Mukherji and Arora, 2017; Schlagwein and Hu, 2017; Sias and Duncan, 2018). Treem and Leonardi (2012) propose four affordances of new technologies that have considerably changed the nature of work and social networking in organizations, namely visibility, persistence, editability, and association. The persistence of an integrated flow of interaction and contextualized associations established between coworkers, in particular, play a crucial role in creating mutual understanding and accumulating social capital among team members. Such findings suggest that norms around the use of such networking technologies must be forged to promote positive communication and avoid potential misunderstanding and conflict.

#### Team Resilience and Adaptability

Alongside research on adaptability and resilience among individuals, there is emerging research on group-level resilience and agility. Resilience at the team-level originates from the interactions between contextual factors (e.g., type of job tasks and culture) and team members (Marks et al., 2001; Stoverink et al., 2018) as individuals collaborate over a period of time (Hartmann et al., 2020). The interpersonal relationships between individuals in a team affect emotional expression and the collective experience of positive emotions, such as shared enthusiasm, optimism, comfort, or relaxation, which foster team resilience (Carmeli et al., 2013; Stephens et al., 2013; Meneghel et al., 2016b). The structure and roles of individuals in a team also influence team resilience (Hartmann et al., 2020). Specifically, team resilience is positively related with in-role and extra-role team performance (Meneghel et al., 2016a,b), with the latter being more important as digital transformation is underway. This is because resilient teams are more likely to find flexible and effective solutions when faced with challenges and adversity.

### Organizational-Level

At the organizational level, we propose three overarching factors in supporting an organization’s digital transformation: leadership; human resources; and organizational culture/climate.

#### Leadership

Leadership is another essential factor that is likely to shape digital transformation processes and outcomes in work teams and organizations and describes a leader’s ability to motivate and influence others to engage in collective activities and accomplish shared goals (Zaccaro and Klimoski, 2002; Yukl, 2006). In general, leadership is found to play a crucial role in organizational functioning and employee performance (Cortellazzo et al., 2019; Gemeda and Lee, 2020).

In an organization’s drive toward digitalization and transformation, leadership, and technological innovations mutually affect each other on an ongoing basis (Cortellazzo et al., 2019). On the one hand, technological advancement poses new challenges and requires leaders to take up new responsibilities and enhance leadership skills according to the changing contexts (Cortellazzo et al., 2019). Specifically, the adoption and implementation of new technologies have been identified as key drivers for initiating disruptive changes in work teams and organizations, which often results in the reconfiguration of established management routines and resistance from members (Bartol and Liu, 2002; Cortellazzo et al., 2019).

In the face of these challenges, leaders are entrusted with a range of emerging responsibilities, including but not limited to creating positive digital cultures, motivating employees to embrace transformation and upskill, and attracting digital experts, among other roles (Roepke et al., 2000; Elenkov et al., 2005; Haddud and McAllen, 2018). In a recent review, Cortellazzo et al. (2019) identified five main skills that characterize effective leadership in the digital era: communicating through digital media, high-speed decision-making, managing disruptive change, managing connectivity, and the renaissance of technical skills. Similarly, responsive leadership, characterized by leaders’ responsiveness to employees’ feedback and continuous leader-employee communication, constitutes an essential skill for leaders in the digital workplace (Dery et al., 2017).

Leadership also influences the direction and outcomes of technology implementation and digital transformation. Recent studies examined the impact of leadership style on workplace innovation based on existing typologies of transformational, transactional and laissez-faire leadership (Bass et al., 2003). In workplace settings, transformational leadership is found to be more effective than transactional and laissez-faire leadership, and predicts better employee performance, job satisfaction, and higher levels of commitment (Bass et al., 2003; Berson and Avolio, 2004; Liao et al., 2010; Gemeda and Lee, 2020). Transformational leadership is also associated with the adoption of technological innovations in organizations (Carreiro and Oliveira, 2019). For example, Carreiro and Oliveira (2019) studied mobile cloud computing adoption and revealed that transformational leadership components, such as vision and personal recognition, were positively related to the firm’s intention to adopt the innovation.

Other studies have highlighted the role of responsive leadership that focuses on employee experiences and connectedness and widespread learning mechanisms (Dery et al., 2017). Responsive leaders encourage experimentation with new technologies and provide opportunities and resources for continuous learning, as discussed below (Dery et al., 2017). For example, studies have highlighted the importance of establishing dedicated units for digital transformation that report directly to senior leaders and/or the CEO and whose role is to drive change throughout the whole organization (Chanias et al., 2019). Similarly, other studies have found that leaders must provide resources and make structural changes to support digital transformation strategic efforts (Matt et al., 2015; Hess et al., 2016). The change process includes reflexive practices by individuals and structural changes such as increased investment in digital technologies, resources, collaboration and new ways of working and practice to support digital transformation (Chanias et al., 2019).

#### Human Resources

Organizations must ensure that their employees have the right skills and competencies as they undergo digital transformation, where human capital resources are one of the most important resources available (Noe et al., 2014). There is increasing recognition of human resource development (HRD) professionals’ role in allocating resources, supporting workplace learning and development, and facilitating organizational change in the context of new technologies (Benson et al., 2002; Li and Herd, 2017; Chuang and Graham, 2018). Case study research maintains that new competencies can be gained through updating existing capabilities through training and new hires or recruiting employees experienced with integration processes or outsourcing hard-to-find skills and competencies (Hess et al., 2016). While the latter two options may be less risky and require less initial investment, a disadvantage is that companies fail to develop competencies internally and may suffer from a lower competitive edge in the future (Hess et al., 2016). Aside from case studies and macro-studies of job automation, there is limited empirical research on the influence of new technologies on employment and related impacts on human resources. However, Chuang and Graham (2018) found that HRD professionals need to urgently increase their knowledge of the impact of technological change on employment and job structures. Priority areas for HRD professionals increased focus on developing human skills and balancing the introduction of new machines and technologies. Moreover, greater understanding of how to transition workers to increasingly skill-polarized work environments, including managing the threat of technological unemployment, is needed (Chuang and Graham, 2018).

The advancement of digital technologies, such as electronic HRM systems and increased HR analytics, is also changing HRD professionals’ role in the context of digitalization (Grant and Newell, 2013; Marler and Fisher, 2013; Marler and Boudreau, 2017). While there is growing attention to the role of e-HRM in allowing HR professionals to enhance their strategic role within organizations (e.g., Grant and Newell, 2013), research on e-HRM is still in an early stage, with limited empirical evidence on whether e-HRM predicts strategic outcomes (Marler and Fisher, 2013). While there is evidence that HRM predicts e-HRM outcomes, this relationship is contextual, with research designs not yet sufficient to establish causal direction (Marler and Fisher, 2013). The literature on HR analytics, defined as HR practices enabled by information technology analytics, benchmarking, and data-driven decision making, is also limited (Marler and Boudreau, 2017). While there is a positive relationship with HR analytics and organizational effectiveness, there is limited scientific evidence to aid decision-making in the adoption of HR analytics. Nonetheless, three moderators may affect the relationship between the adoption of HR analytics and organizational outcomes, including HR professional analytics skills, managerial buy-in, and the integration of HR information technology. For example, current challenges include both the quality and accessibility of e-HRM software systems and HR capabilities in analyzing and interpreting data (Marler and Boudreau, 2017).

## Organizational Culture/Climate

There is growing recognition of the role of organizational culture in digital transformation (Hartl and Hess, 2017; Osmundsen et al., 2018); yet, few studies have examined this empirically. Case study research has, however, found that traditional command and control structures often reinforce work-group silos and make it much harder for employees to respond rapidly to customer demands and needs (Dery et al., 2017). Instead, alongside top-down transformation efforts, including clear task and role descriptions of senior leaders, bottom-up strategies such as employee engagement are important in digital transformation and innovation (Dery et al., 2017; Chanias et al., 2019). Key initiatives include engaging internal actors in “episodes of digital strategy making” (Chanias et al., 2019, p. 30). Specifically, leaders and managers can initiate cultural change through various communication measures, such as all staff emails, workshops, “fireside chats,” and promotional materials (Chanias et al., 2019, p. 25). The engagement of internal stakeholders and representatives across different organizational departments through communications, such as videos, manuals, posters, ideas, and workshops for employees on new digital technologies, helped facilitate the change process (Chanias et al., 2019). Developing concept pitches and prototypes through internal and external channels (e.g., employees pitching for ideas) positively impacted the organization and showed a higher possibility for digital innovation than previously anticipated by leaders (Chanias et al., 2019). Mueller and Renken (2017) found that communication and collaboration technology enabled a digitally enabled workplace and supported process innovation. In particular, alignment with IT-processes, including internal communication and marketing and employees’ involvement, helped them reinvent and reimagine their work (Mueller and Renken, 2017). As discussed above, collaborative technologies, including social media platforms, can promote innovation and develop open and entrepreneurial cultures (Dery et al., 2017; Chanias et al., 2019). However, key challenges include resistance by senior leaders and managers and conflicts between departments on digital transformation plans and processes. The slow pace of change and leadership and employee turnover were also cited as key challenges (Chanias et al., 2019).

While case study research has revealed important insights into digital transformation processes, more rigorous integration of existing theoretical and empirical frameworks are needed. Organizational culture is defined as a pattern of shared assumptions, beliefs, values, and norms learned by a group and taught to new members (Schein, 2004). The study of organizational culture has a long trajectory within anthropology, sociology, and social psychology (Hartnell et al., 2011). While organizational culture has been traditionally studied using qualitative methods such as ethnography, survey-based methods have become more dominant in recent decades (Schneider et al., 2013; Denison et al., 2014).

Recent reviews have focused on the link between organizational culture and employee and organizational processes and outcomes (Hartnell et al., 2011; Denison et al., 2014). The Competing Values Framework (CVF) is one of the most highly utilized organizational culture measures and theorizes that four different culture types exist across two opposing value systems: flexibility versus control and internal versus external orientation (Quinn and Rohrbaugh, 1983). These relate to organizational effectiveness indicators due to their underlying assumptions, beliefs, values, and artifacts. For instance, a “clan” based culture, which prioritizes human resources and affiliation, can be linked to employee effectiveness criteria such as employee satisfaction and commitment. Meanwhile, an “adhocracy” culture, which relies on risk-taking, creativity, and adaptability, can be linked to innovation outcomes (Hartnell et al., 2011).

Generally, meta-reviews have found that CVF’s culture types are significantly associated with organizational effectiveness (Hartnell et al., 2011). While all culture types had moderate to strong associations with operational effectiveness, job satisfaction was notably higher in organizations with clan cultures (i.e., family like, collaborative organizations) than adhocracy and market cultures. However, market culture was more strongly associated with subjective innovation, quality of products and services, and financial effectiveness (Hartnell et al., 2011). Another meta-review of the CVF found that organizational culture is an important factor in driving innovation (Büschgens et al., 2013). Managers of innovative organizations were more likely to implement a developmental culture, emphasizing an external and flexibility orientation that is largely consistent with an innovative organization’s goals. On the other hand, hierarchical cultures that emphasized control and internal orientation were less likely to be found in innovative organizations (Büschgens et al., 2013). Nonetheless, regardless of orientation, it is important to align innovation strategy with organizational cultural values to ensure its effectiveness (Büschgens et al., 2013), where other studies have confirmed the fit between organizational culture and innovation strategy (Chen et al., 2018).

In a study of digitalization experts, Hartl and Hess (2017) reported that experts highlighted flexible (i.e., clan/adhocracy) over control (i.e., hierarchical/market) organizational cultures as critical to digital transformation success. In digital transformation, cultures that promoted values such as openness toward change, agility, a tolerance toward failure, and a willingness to learn were more valued. Innovation, risk affinity, and entrepreneurship alongside cooperation, community, and customer-centricity were also cited as important organizational values. Another study conducted with company stakeholders found that organizations can develop digital cultures, break down resistance to digitalization and cultivate transparent-oriented cultures by adopting strategies such as reverse mentoring to improve digital competencies and skills (Brunetti et al., 2020).

Organizational climate is a related yet distinct concept to organizational culture and is defined as employee perceptions of policies, practices, and employee experiences, along with behaviors that employees observe as being rewarded and supported (Ostroff et al., 2003; Schneider et al., 2013). Organizational climate can be both a global concept (Jung et al., 2003) or linked to more narrow strategic goals (Zohar and Hofmann, 2012). Nonetheless, organizational culture and climate overlap, with commonly used climate measures developed from existing culture constructs such as the CVF (Patterson et al., 2005). Establishing the level at which perception data is collected and analyzed (e.g., individual versus group versus organizational) plays a vital role in organizational climate research (Zohar and Hofmann, 2012; Schneider et al., 2013). In general, studies have shown positive climate-performance relationships. A recent meta-analysis by Beus et al. (2020) integrating the CVF found positive climate–performance associations for different climate types, with job attitudes as a common mediator. Transformational leadership, innovative work behavior, and LMX-exchange have been linked to higher innovation climate (Aarons and Sommerfeld, 2012), while innovative work behavior played a mediating role in the relationship between organizational climate for innovation and organizational performance in other studies (Shanker et al., 2017).

Additionally, transformational leadership and climate in organizations foster adaptive performance in workers (Charbonnier-Voirin et al., 2010). In a multilevel analysis, Charbonnier-Voirin et al. (2010) found a positive relationship between transformational leadership and adaptive performance at the individual level, while team-level transformational climate exerted positive cross-level effects on adaptive performance. Finally, team-level climate for innovation moderated the relationship of individual perceptions of transformational leadership with adaptive performance. Shipton et al. (2005) found that effective HRM systems predicted product and production technology innovation and that innovation was more enhanced when there was a supportive learning climate but inhibited when there is a link between appraisal and remuneration. Overall, these findings highlight the importance of culture and climate to other individual, group, and organizational factors examined in this review.

## Summary of Findings and Discussion; a Multi-Level Framework for Digital Transformation

**FIGURE 1:**
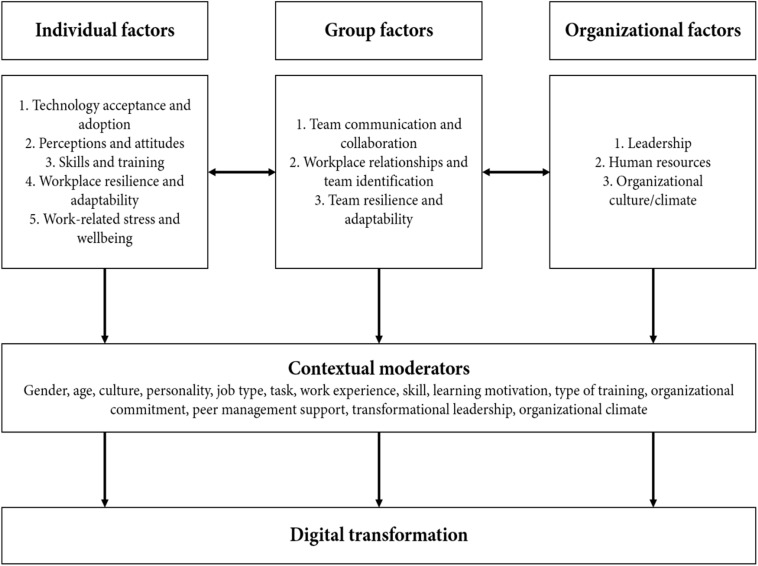
A multi-level theoretical framework for understanding workplace digital transformation.

Our review sought to identify important factors for workplace digital transformation and present them in a multi-level framework. The framework (see Figure 1) integrates identified factors with potential moderators at the individual, group, and organizational levels. Specifically, we married studies on digitalization and digital transformation with existing models of organizational behavior and management (e.g., Ployhart, 2015; Robbins and Judge, 2019). By so doing, this work bridges existing gaps in the digital transformation research literature that has primarily focused at the technology and business level (e.g., Verhoef et al., 2019; Vial, 2019) with less integration of employee, work-group and organizational factors.

At the individual level, we theorized that five factors related to effective digital transformation among employees: technology acceptance and adoption; perception and attitudes toward technology and digital transformation; skills and training; workplace resilience and adaptability, and work-related wellbeing. At the group-level, we identified three factors necessary for digital transformation: team communication and collaboration; workplace relationships and team identification, and team adaptability and resilience. Finally, at the organizational-level, we proposed three factors for digital transformation: leadership; human resources, and organizational climate/culture. Our review of the literature suggests that these factors are important to be considered when planning for and embarking on digital transformation. Nevertheless, there is evidence that specific digital transformation outcomes may be moderated by a host of personal, contextual and cultural moderators, which should be taken into account when implementing digital transformation. While in this review and in the framework summarizing our findings we have added an expanded list of these moderators for reference, in reality they might not be present or relevant simultaneously. More research is needed to understand the role of moderating factors in digital transformation. Following this synthesis, we discuss the implications of our findings for further research and practice.

As the introduction of digital technologies is often the cornerstone of digital transformation in the workplace, it is critical that acceptance and attitudes of employers toward new technologies fosters its adoption and consequently facilitates digital transformation plans. Our review identified that if employees perceive that a particular technology or system will be useful to their work and will help them to perform well, and is easy for them to learn and use, they are more likely to accept it. Additionally, we found that technology adoption differs by contextual factors, such as age, gender task-technology fit, and prior work experience. Technological adoption and acceptance is also associated with resilience and opportunities for training. Peer and top management support influence technology adoption at the group and organizational levels. In general, studies showed that employees are generally motivated to support new technologies and see benefits such as enhanced productivity and work quality, however, attitudes and perceptions are moderated by occupation, job role, gender, age and technology type. For example, when technology was perceived as leading to job loss or reductions, attitudes were negative and related to increased turnover, cynicism, depression, lower organizational commitment and career satisfaction. Nevertheless, perceived organizational support and competitive psychological climate helped to moderate negative perceptions and outcomes. Employee expectations of autonomy, competence, and engagement were also linked to increased support for digital transformation.

Skills upgrading or retraining are also important precursors of digital transformation as studies have shown that employees need a mix of cognitive, technical digital skills in increasingly digital work environments. However, it can be a practical challenge to motivate employees to do so. We found that factors such as learning motivation, attitudes, personality, and skill-levels at the individual level are likely to moderate learning outcomes and the transfer of training to practice. In addition, co-workers’ attitudes, supervisor and peer support, being able to volunteer for training instead of being mandated to, and the extent to which employees are involved in the design of training programs are also important factors to consider in the transfer of skills and training at the group and organizational levels. Developing skills and providing adequate training is an urgent imperative as organizations undergo digital transformation. Prior research on the role of individual factors, such as cognitive ability and motivation, alongside peer, supervisor and team support for training, can help companies to develop and refine training programs, ensure that adequate resources are provided for training, and create personalized training opportunities that cater to different employee needs.

Digital transformations in workplaces can be a period of change and uncertainty for individuals and organizations alike. Thus, it is highly likely that individual resilience and adaptability in the workplace will be key traits for seamless digital transformation, however, these have not been well studied at present. Existing theory and research have shown that workplace resilience is related to job satisfaction and performance, organizational citizenship behavior and commitment, work engagement, openness, and commitment to organizational change and behavioral adaptation. Adaptability, a related concept, suggests that adaptable workers will be more successful during digital transformation as they are more proactive and take responsibility for adjusting to changing situations. As with resilience, personality traits such as openness to experience, emotional stability, conscientiousness, and ambition are positively related to individuals’ adaptive performance and are relevant as digital transformation takes place.

Due to evidence that digital technology contributes to increasing stress and fragmentation and blurring of work-life boundaries, employers will need to employ strategies to mitigate these detrimental impacts on employee well-being and engagement. A key area of focus could include programs and training to foster workplace resilience and adaptability and cultivate a mindset shift in being adaptable in the context of ongoing job and digital disruption. Technostress may be increasingly salient in digitalization and digital transformation, leading to increasing fragmentation and blurring of work-life boundaries, which can lower productivity, well-being, and organizational commitment. New technologies can also exacerbate other occupational stressors such as work overload and lack of control, especially among managers but could also yield positive outcomes at work, including increased effectiveness and innovation.

The nature of work might also evolve as digital technology is introduced and work processes evolve. However, working in teams and collaborating across teams is likely to remain essential to organizational functioning and the quality of collaborative work practices. These are in turn linked to higher levels of innovative performance in work teams and organizations. As new digital communication tools are introduced in workplaces, it is necessary to ensure that they facilitate information flow, ideas, and task integration to enhance collaboration rather than adding unnecessary complexity to the process. The rapid increase in the ability for teams to work virtually across technology platforms certainly facilitate both task-oriented and relational communication among team members and lead to positive outcomes, such as more efficient knowledge sharing and information flow, more precise task coordination, and increased transparency, while flexible and cross-functional teams can also facilitate collaboration and support digital transformation goals. Other structural mechanisms, such as digital hubs and internal digital experts further support innovation and digital solutions among teams. Conversely, a lack of collaboration and communication can impede digital transformation efforts and lead to resistance.

Despite the increased adoption of technology in workplaces, the quality and style of workplace relationships will continue to be important to support workplace transformation. Specifically, high-quality supervisor-subordinate relationships and team-members’ exchange positively promote innovative work behaviors, while misalignment between new technologies and established team identities can lead to resistance. Indeed, the growth of multi-functional management and communication technologies provides new opportunities for employee and team engagement and interactions. This also fosters adaptable and resilient teams and build stronger team identification, which bodes well for thriving amidst challenges and adversity during digital transformations.

Organizational leaders continue to be essential in leading change, including motivating employees to embrace digital transformation. The adoption and implementation of new technologies is likely to disrupt established structures and routines, which will in turn cause uncertainty and resistance.

Therefore, transformational leadership styles may be more effective in digital transformation than transactional and laissez-faire leadership, due to more positive outcomes in employee performance, job satisfaction, and organizational commitment. Leaders who are responsive to employee experiences and encourage experimentation may also be more effective in leading digital transformation.

Alongside the role of leaders, digital transformation is creating expanded roles for human resource professionals. Priority areas include increasing knowledge of recruiting, retaining, reskilling, and transitioning workers in increasingly skill-polarized work environments and developing positive organizational culture, including in relation to learning. HR professionals can also focus on enhanced used of e-HRM systems and HR analytics to strengthen their strategic roles. Finally, organizational culture and climate are likely to shape digital transformation processes and outcomes in the workplace. Specifically, there is evidence that traditional command and control structures reinforce work-group silos and make it much harder for employees to respond rapidly to customer demands. Instead, bottom-up engagement in digital strategy and change supports digital transformation and innovation. More research is needed, however, to understand the role of organizational climate and culture in shaping digital transformation. On the whole, these findings and the framework presented here are relevant for organizations and managers as they digitalize and embark on digital transformation.

### Directions for Future Research

This study has some limitations but also presents several opportunities for further research. Our review is broad in scope and integrates qualitative and non-qualitative studies using varying research designs rather than being a systematic review. We opted for this targeted approach as the field of digital transformation is multi-disciplinary and still in its nascent stages, thus limiting the potential and usefulness of systematic reviews and meta-analyses. While we have integrated an expansive set of literature into a framework that links individual, group, and organizational factors to digital transformation processes and outcomes, further research is needed to test these hypotheses and relationships. The studies included in the reviews were also largely cross-sectional studies that used self-report measures that provide useful insights at one particular time point but have limited value in understanding change processes, which longitudinal or qualitative studies are better suited for.

Nevertheless, a key contribution of this review is the integration of several under-studied individual, group, and organizational factors into a holistic, multi-level digital transformation framework. For example, technology adoption has been studied extensively and in a wide range of workplace settings. As the uptake of new technologies increases due to rapid digitalization, we propose that other research further integrates the rich body of literature on technology adoption with digital transformation processes and outcomes. This proposed framework provides researchers and practitioners with a useful overview of the body of knowledge that exists today and a reference for identifying either areas for future research or issues to focus on when embarking on digital transformation.

This review has highlighted the importance of context at the individual, group, and organizational levels. At the individual level, factors such as gender, age, personality, education, job type/job tasks, and experience/skill levels are all likely to play a role in digital transformation outcomes, such as job satisfaction, productivity, and task performance, alongside work-related wellbeing, and stress, organizational commitment and turnover. Social norms and peer and management support may influence group outcomes such as team effectiveness, empowerment and participation, resilience, and adaptability. At the organizational level, leadership, organizational culture and climate are likely to influence digital transformation outcomes, yet may be moderated by factors such as human resource management, support for training, and organizational setting. Future research should test these relationships, including more study of noted contextual factors to draw out relevant industry and policy findings. Many of the factors included in our review occur at multiple organizational levels, with some overlap of concepts across different levels. Therefore, more attention is needed to clarify the relationships between different factors at multiple organizational levels.

Lastly, existing reviews and digital transformation studies have mostly focused on strategic or business level processes, with scant attention to employee-related factors at the individual, group, and organizational levels. For instance, existing digital transformation research has focused mainly on executives and organizational leaders’ perspectives rather than those of employees. Therefore, a priority for future research includes further study of employee attitudes and perceptions of digital transformation, given that employee perceptions are likely to differ from those of managers.

## Conclusion

The rapid advancement of new digital technologies in the workplace is inevitable and will lead to transformation across the economy while increasing concerns about the future of work among organizations and their workers. Organizations need to embrace digital technologies and transform in order to remain competitive and survive. Employees are a crucial part of the digital transformation process’s success and understanding their perceptions and attitudes toward technological change is important, alongside other strategies to enhance their digital capabilities. This review distilled the important factors in digital transformation at three different levels (individual, group and organization) to highlight the crucial role that employees, organizational leaders, managers, and human resource departments play in this transformation process. Organizations and their leaders also need to be mindful of the unintended adverse effects of technological change and digital transformation on employees and mitigate impacts on work-related health and well-being through promoting resilience and adaptability among individuals and teams with requisite support.

## Author Contributions

BT and SC conceptualized the review. BT performed the bibliographic search and prepared the first draft of the manuscript. SC helped develop this review and the first version of this manuscript. YW, ZS, HL, and PO contributed to the selection of literature and the results. SL helped develop this review and edited the full manuscript. All authors contributed to and approved the final manuscript.

## Conflict of Interest

The authors declare that the research was conducted in the absence of any commercial or financial relationships that could be construed as a potential conflict of interest.

## References

[B1] Aarons G. A. Sommerfeld D. H. (2012). Leadership, innovation climate, and attitudes toward evidence-based practice during a statewide implementation. *J. Am. Acad. Child Adolesc. Psychiatry* 51 423–431. 10.1016/j.jaac.2012.01.018 22449648 PMC3841107

[B2] Acemoglu D. Autor D. (2011). “Skills, tasks and technologies: implications for employment and earnings,” in *Handbook of Labor Economics*, eds Ashenfelter O. Card D. E. (Amsterdam: Elsevier), 1043–1171.

[B3] Agarwal R. Gao G. DesRoches C. Jha A. K. (2010). The digital transformation of healthcare: current status and the road ahead. *Inf. Syst. Res.* 21 796–809. 10.1287/isre.1100.0327 19642375

[B4] Agarwal Upasna A. Datta S. Blake−Beard S. Bhargava S. (2012). Linking LMX, innovative work behaviour and turnover intentions: the mediating role of work engagement. *Career Dev. Int* 17 208–230. 10.1108/13620431211241063

[B5] Ajzen I. (1985). “From intentions to actions: a theory of planned behavior,” in *Action Control: From Cognition to Behavior*, eds Kuhl J. Beckmann J. (Berlin: Springer), 11–39.

[B6] Ajzen I. (1991). The theory of planned behavior. *Organ. Behav. Hum. Decis. Process.* 50 179–211. 10.1016/0749-5978(91)90020-T

[B7] Alshawi M. Ingirige B. (2003). Web-enabled project management: an emerging paradigm in construction. *Autom. Constr.* 12 349–364. 10.1016/s0926-5805(03)00003-7

[B8] Anders A. (2016). Team communication platforms and emergent social collaboration practices. *Int. J. Bus. Commun.* 53 224–261. 10.1177/2329488415627273

[B9] Arntz M. Gregory T. Zierahn U. (2017). Revisiting the risk of automation. *Econ. Lett.* 159 157–160. 10.1016/j.econlet.2017.07.001

[B10] Atitumpong A. Badir Y. F. (2018). Leader-member exchange, learning orientation and innovative work behavior. *J. Workplace. Learn.* 30 32–47. 10.1108/JWL-01-2017-0005

[B11] Avolio B. J. Bass B. M. Jung D. I. (1999). Re-examining the components of transformational and transactional leadership using the multifactor leadership questionnaire. *J. Occup. Organ. Psychol.* 72 441–462. 10.1348/096317999166789

[B12] Ayyagari R. Grover V. Purvis R. (2011). Technostress: technological antecedents and implications. *Manag. Inf. Syst. Q.* 35 831–858. 10.2307/41409963

[B13] Baard S. K. Rench T. A. Kozlowski S. W. J. (2014). Performance adaptation: a theoretical integration and review. *J. Manag.* 40 48–99. 10.1177/0149206313488210

[B14] Badran M. A. Youssef-Morgan C. M. (2015). Psychological capital and job satisfaction in Egypt. *J. Manag. Psychol.* 30 354–370. 10.1108/JMP-06-2013-0176

[B15] Bakker A. B. Demerouti E. (2007). The job demands−resources model: state of the art. *J. Manag. Psychol.* 22 309–328. 10.1108/02683940710733115

[B16] Bakker A. B. Demerouti E. (2017). Job demands-resources theory: taking stock and looking forward. *J. Occup. Health Psychol.* 22 273–285. 10.1037/ocp0000056 27732008

[B17] Bakker A. B. Demerouti E. ten Brummelhuis L. (2012). Work engagement, performance, and active learning: the role of conscientiousness. *J. Vocat. Behav.* 80 554–564. 10.1016/j.jvb.2011.08.008

[B18] Bandura A. (1986). *Social Foundations of Thought and Action: A Social Cognitive Theory.* Englewood Cliffs, NJ: Prentice-Hall, Inc.

[B19] Banker R. D. Bardhan I. Asdemir O. (2006). Understanding the Impact of collaboration software on product design and development. *Inf. Syst. Res.* 17 352–373. 10.1287/isre.1060.0104 19642375

[B20] Bartol K. M. Liu W. (2002). “Information technology and human resources management: harnessing the power and potential of netcentricity,” in *Research in Personnel and Human Resources Management*, eds Buckley M. Halbesleben J. Wheeler A. R. (Bingley: Emerald Group Publishing Limited), 215–242.

[B21] Bass B. M. Avolio B. J. Jung D. I. Berson Y. (2003). Predicting unit performance by assessing transformational and transactional leadership. *J. Appl. Psychol.* 88 207–218. 10.1037/0021-9010.88.2.207 12731705

[B22] Beer P. Mulder R. H. (2020). The effects of technological developments on work and their implications for continuous vocational education and training: a systematic review. *Front. Psychol.* 11:918. 10.3389/fpsyg.2020.00918 32457688 PMC7226038

[B23] Benson A. D. Johnson S. D. Kuchinke P. K. (2002). The use of technology in the digital workplace: a framework for human resource development. *Adv. Dev. Hum. Resour.* 4 392–404. 10.1177/152342202237518

[B24] Berg S. A. Chyung S. Y. (2008). Factors that influence informal learning in the workplace. *J. Workplace Learn.* 20 229–244. 10.1108/13665620810871097

[B25] Berghaus S. Back A. (2016). Stages in digital business transformation: results of an empirical maturity study. *Paper presented at the 10th Mediterranean Conference on Information Systems*, Paphos.

[B26] Berman S. J. (2012). Digital transformation: opportunities to create new business models. *Strategy Leadersh.* 40 16–24. 10.1108/10878571211209314

[B27] Berson Y. Avolio B. J. (2004). Transformational leadership and the dissemination of organizational goals: a case study of a telecommunication firm. *Leadersh. Q.* 15 625–646. 10.1016/j.leaqua.2004.07.003

[B28] Besson P. Rowe F. (2012). Strategizing information systems-enabled organizational transformation: a transdisciplinary review and new directions. *J. Strateg. Inf. Syst.* 21 103–124. 10.1016/j.jsis.2012.05.001

[B29] Beus J. M. Solomon S. J. Taylor E. C. Esken C. A. (2020). Making sense of climate: a meta-analytic extension of the competing values framework. *Organ. Psychol. Rev.* 10 136–168. 10.1177/2041386620914707

[B30] Blease C. Bernstein M. H. Gaab J. Kaptchuk T. J. Kossowsky J. Mandl K. D. (2018). Computerization and the future of primary care: a survey of general practitioners in the UK. *PLoS One* 13:e0207418. 10.1371/journal.pone.0207418 30540791 PMC6291067

[B31] Blume B. D. Ford J. K. Baldwin T. T. Huang J. L. (2010). Transfer of training: a meta-analytic review. *J. Manag.* 36 1065–1105. 10.1177/0149206309352880

[B32] Bode E. Gold R. (2018). Adult training in the digital age. *Economics* 12 1–14. 10.5018/economics-ejournal.ja.2018-36

[B33] Bolívar-Ramos M. García-Morales V. García-Sánchez E. (2012). Technological distinctive competencies and organizational learning: effects on organizational innovation to improve firm performance. *J. Eng. Technol. Manag.* 29 331–357. 10.1016/j.jengtecman.2012.03.006

[B34] Bolstad C. A. Endsley M. R. (2003). Tools for supporting team collaboration. *Paper Presened at the 47th Annual Meeting of the*, Santa Monica, CA: Human Factors and Ergonomics Society.

[B35] Bond M. Marín V. I. Dolch C. Bedenlier S. Zawacki-Richter O. (2018). Digital transformation in German higher education: student and teacher perceptions and usage of digital media. *Int. J. Educ. Technol. High. Educ.* 15:48. 10.1186/s41239-018-0130-1

[B36] Bondarouk T. Parry E. Furtmueller E. (2017). Electronic HRM: four decades of research on adoption and consequences. *Int. J. Hum. Resour. Manag.* 28 98–131. 10.1080/09585192.2016.1245672

[B37] Börner K. Scrivner O. Gallant M. Ma S. Liu X. Chewning K. (2018). Skill discrepancies between research, education, and jobs reveal the critical need to supply soft skills for the data economy. *Proc. Natl. Acad. Sci. U.S.A.* 115 12630–12637. 10.1073/pnas.1804247115 30530667 PMC6294902

[B38] Bouckenooghe D. Raja U. Butt A. N. (2013). Combined effects of positive and negative affectivity and job satisfaction on job performance and turnover intentions. *J. Psychol.* 147 105–123. 10.1080/00223980.2012.678411 23469474

[B39] Boughzala I. de Vreede G.-J. (2015). Evaluating team collaboration quality: the development and field application of a collaboration maturity model. *J. Manag. Inf. Syst*. 32 129–157. 10.1080/07421222.2015.1095042

[B40] Boughzala I. de Vreede G.-J. Limayem M. (2012). Team collaboration in virtual worlds: editorial to the special issue. *J. Assoc. Inf. Syst.* 13 714–734. 10.17705/1jais.00313

[B41] Bowling N. A. Eschleman K. J. Wang Q. (2010). A meta-analytic examination of the relationship between job satisfaction and subjective well-being. *J. Occup. Organ. Psychol.* 83 915–934. 10.1348/096317909X478557

[B42] Briggs R. O. de Vreede G.-J. Nunamaker J. F. J. (2003). Collaboration engineering with think lets to pursue sustained success with group support systems. *J. Manag. Inf. Syst.* 19 31–64. 10.1080/07421222.2003.11045743

[B43] Britt T. W. Shen W. Sinclair R. R. Grossman M. R. Klieger D. M. (2016). How much do we really know about employee resilience? *Ind. Organ. Psychol.* 9 378–404. 10.1017/iop.2015.107

[B44] Brougham D. Haar J. (2017). Employee assessment of their technological redundancy. *Lab. Ind.* 27 213–231. 10.1080/10301763.2017.1369718

[B45] Brougham D. Haar J. (2018). Smart technology, artificial intelligence, robotics, and algorithms (STARA): employees’ perceptions of our future workplace. *J. Manag. Organ.* 24 239–257. 10.1017/jmo.2016.55

[B46] Brown P. Souto-Otero M. (2020). The end of the credential society? An analysis of the relationship between education and the labour market using big data. *J. Educ. Policy.* 35 95–118. 10.1080/02680939.2018.1549752

[B47] Brown S. A. Massey A. P. Montoya-weiss M. M. Burkman J. R. (2002). Do I really have to? User acceptance of mandated technology. *Eur. J. Inf. Syst.* 11 283–295. 10.1057/palgrave.ejis.3000438

[B48] Brunetti F. Matt D. T. Bonfanti A. De Longhi A. Pedrini G. Orzes G. (2020). Digital transformation challenges: strategies emerging from a multi-stakeholder approach. *TQM J.* 32 697–724. 10.1108/tqm-12-2019-0309

[B49] Brynjolfsson E. McAfee A. (2014). *The Second Machine Age: Work, Progress, and Prosperity in a Time of Brilliant Technologies.* New York, NY: W. W. Norton and Company.

[B50] Burns E. Poikkeus A.-M. Aro M. (2013). Resilience strategies employed by teachers with dyslexia working at tertiary education. *Teach. Teach. Educ.* 34 77–85. 10.1016/j.tate.2013.04.007

[B51] Burton-Jones A. Akhlaghpour S. Ayre S. Barde P. Staib A. Sullivan C. (2020). Changing the conversation on evaluating digital transformation in healthcare: insights from an institutional analysis. *Inf. Organ.* 30:100255. 10.1016/j.infoandorg.2019.100255

[B52] Burton-Jones A. Gallivan M. J. (2007). Toward a deeper understanding of system usage in organizations: a multilevel perspective. *Manag. Inf. Syst. Q.* 31 657–679. 10.2307/25148815

[B53] Burton-Jones A. Hubona G. S. (2006). The mediation of external variables in the technology acceptance model. *Inf. Manag.* 43 706–717. 10.1016/j.im.2006.03.007

[B54] Büschgens T. Bausch A. Balkin D. B. (2013). Organizational culture and innovation: a meta-analytic review. *J. Prod. Innov. Manag.* 30 763–781. 10.1111/jpim.12021

[B55] Cadwallader S. Jarvis C. B. Bitner M. J. Ostrom A. L. (2010). Frontline employee motivation to participate in service innovation implementation. *J. Acad. Mark. Sci.* 38 219–239. 10.1007/s11747-009-0151-3

[B56] Cameron F. Brownie S. (2010). Enhancing resilience in registered aged care nurses. *Australas. J. Ageing* 29 66–71. 10.1111/j.1741-6612.2009.00416.x 20553536

[B57] Carmeli A. Friedman Y. Tishler A. (2013). Cultivating a resilient top management team: the importance of relational connections and strategic decision comprehensiveness. *Saf. Sci.* 51 148–159. 10.1016/j.ssci.2012.06.002

[B58] Carreiro H. Oliveira T. (2019). Impact of transformational leadership on the diffusion of innovation in firms: application to mobile cloud computing. *Comput. Ind.* 107 104–113. 10.1016/j.compind.2019.02.006

[B59] Cascio W. F. (2019). Training trends: macro, micro, and policy issues. *Hum. Resour. Manag. Rev.* 29 284–297. 10.1016/j.hrmr.2017.11.001

[B60] Cascio W. F. Montealegre R. (2016). How technology is changing work and organizations. *Annu. Rev. Organ. Psychol. Organ. Behav.* 3 349–375. 10.1146/annurev-orgpsych-041015-062352

[B61] Chan D. (2019). Team-level constructs. *Annu. Rev. Organ. Psychol. Organ. Behav.* 6 325–348. 10.1146/annurev-orgpsych-012218-015117

[B62] Chanias S. Myers M. D. Hess T. (2019). Digital transformation strategy making in pre-digital organizations: the case of a financial services provider. *J. Strateg. Inf. Syst.* 28 17–33. 10.1016/j.jsis.2018.11.003

[B63] Chao G. T. Kozlowski S. W. (1986). Employee perceptions on the implementation of robotic manufacturing technology. *J. Appl. Psychol.* 71 70–76. 10.1037/0021-9010.71.1.70

[B64] Charbonnier-Voirin A. El Akremi A. Vandenberghe C. (2010). A multilevel model of transformational leadership and adaptive performance and the moderating role of climate for innovation. *Group Organ. Manag.* 35 699–726. 10.1177/1059601110390833

[B65] Chauhan R. Ghosh P. Rai A. Shukla D. (2016). The impact of support at the workplace on transfer of training: a study of an Indian manufacturing unit. *Int. J. Train. Dev.* 20 200–213. 10.1111/ijtd.12083

[B66] Chen Z. Huang S. Liu C. Min M. Zhou L. (2018). Fit between organizational culture and innovation strategy: implications for innovation performance. *Sustainability* 10:3378. 10.3390/su10103378

[B67] Chuang S. Graham C. M. (2018). Embracing the sobering reality of technological influences on jobs, employment and human resource development: a systematic literature review. *Eur. J. Train. Dev.* 42 400–416. 10.1108/ejtd-03-2018-0030

[B68] Chuttur M. (2009). “Overview of the Technology Acceptance Model: Origins, Developments and Future Directions,” in *Sprouts: Working papers on Information Systems*, (USA: Indiana University), 9.

[B69] Cole M. S. Schaninger W. S. J. Harris S. G. (2002). The workplace social exchange network: a multilevel, conceptual examination. *Group Organ. Manag.* 27 142–167. 10.1177/1059601102027001008

[B70] Compeau D. Higgins C. (1995). Computer self-efficacy: development of a measure and initial test. *Manag. Inf. Syst.* Q. 19 189–211. 10.2307/249688

[B71] Cortellazzo L. Bruni E. Zampieri R. (2019). The role of leadership in a digitalized world: a review. *Front. Psychol* 10:1938. 10.3389/fpsyg.2019.01938 31507494 PMC6718697

[B72] Cullen K. L. Edwards B. D. Casper W. C. Gue K. R. (2014). Employees’ adaptability and perceptions of change-related uncertainty: implications for perceived organizational support, job satisfaction, and performance. *J. Bus. Psychol.* 29 269–280. 10.1007/s10869-013-9312-y

[B73] Danna K. Griffin R. W. (1999). Health and Well-Being in the Workplace: a review and synthesis of the literature. *J. Manag.* 25 357–384. 10.1177/014920639902500305

[B74] Davis F. (1989). Perceived usefulness, perceived ease of use, and user acceptance of information technology. *Manag. Inf. Syst. Q.* 13 319–340. 10.2307/249008

[B75] Davis F. Bagozzi R. Warshaw P. (1989). User acceptance of computer technology: a comparison of two theoretical models. *Manag. Sci.* 35 982–1003. 10.1287/mnsc.35.8.982 19642375

[B76] Davis F. D. Bagozzi R. P. Warshaw P. R. (1992). Extrinsic and intrinsic motivation to use computers in the workplace. *J. Appl. Soc. Psychol.* 22 1111–1132. 10.1111/j.1559-1816.1992.tb00945.x

[B77] Denison D. Nieminen L. Kotrba L. (2014). Diagnosing organizational cultures: a conceptual and empirical review of culture effectiveness surveys. *Eur. J. Work Organ. Psychol.* 23 145–161. 10.1080/1359432X.2012.713173

[B78] Dery K. Sebastian I. M. van der Meulen N. (2017). The digital workplace is key to digital innovation. *MIS Q. Exec*. 16 135–152.

[B79] Dewett T. Jones G. R. (2001). The role of information technology in the organization: a review, model, and assessment. *J. Manag.* 27 313–346. 10.1177/014920630102700306

[B80] Di Pietro L. Pantano E. Di Virgilio F. (2014). Frontline employees’ attitudes towards self-service technologies: threats or opportunity for job performance? *J. Retail. Consum. Serv.* 21 844–850. 10.1016/j.jretconser.2014.02.014

[B81] Diener E. Lucas R. Oishi S. (2018). Advances and open questions in the science of subjective well-being. *Collabra. Psychol.* 4:15. 10.1525/collabra.115 30637366 PMC6329388

[B82] Doraiswamy P. M. Narayan V. A. Manji H. K. (2018). Mobile and pervasive computing technologies and the future of Alzheimer’s clinical trials. *NPJ Digit. Med.* 1:1. 10.1038/s41746-017-0008-y 31304287 PMC6550135

[B83] Dutta P. Borah A. S. (2018). A study on role of moderating variables in Influencing employees’ acceptance of information technology. *Vis. J. Bus. Perspect*. 22 387–394. 10.1177/0972262918803467

[B84] Ederer P. Nedelkoska L. Patt A. Castellazzi S. (2015). What do employers pay for employees’ complex problem solving skills? *Int. J. Lifelong Educ*. 34 430–447. 10.1080/02601370.2015.1060026

[B85] Edmans A. (2012). The link between job satisfaction and firm value, with implications for corporate social responsibility. *Acad. Manag. Perspect.* 26 1–19. 10.5465/amp.2012.0046

[B86] Elenkov D. Judge W. Wright P. (2005). Strategic leadership and executive innovation influence: an international multi-cluster comparative Ssudy. *Strateg. Manag. J.* 26 665–682. 10.1002/smj.469

[B87] Ellison N. B. Gibbs J. L. Weber M. S. (2014). The use of enterprise social network sites for knowledge sharing in distributed organizations. *Am. Behav. Sci.* 59 103–123. 10.1177/0002764214540510

[B88] Faems D. Van Looy B. Debackere K. (2005). Interorganizational collaboration and innovation: toward a portfolio approach. *J. Prod. Innov. Manag.* 22 238–250. 10.1111/j.0737-6782.2005.00120.x

[B89] Fay M. J. Kline S. L. (2011). Coworker relationships and informal communication in high-intensity telecommuting. *J. Appl. Commun. Res.* 39 144–163. 10.1080/00909882.2011.556136

[B90] Ferris P. A. Sinclair C. Kline T. J. (2005). It takes two to tango: personal and organizational resilience as predictors of strain and cardiovascular disease risk in a work sample. *J. Occup. Health Psychol*. 10 225–238. 10.1037/1076-8998.10.3.225 16060726

[B91] Field J. Chan X. W. (2018). Contemporary knowledge workers and the boundaryless work-life interface: implications for the human resource management of the knowledge workforce. *Front. Psychol* 9:2414. 10.3389/fpsyg.2018.02414 30555399 PMC6283975

[B92] Fisher C. D. (2003). Why do lay people believe that satisfaction and performance are correlated? Possible sources of a commonsense theory. *J. Organ. Behav*. 24 753–777. 10.1002/job.219

[B93] Fisher D. M. Ragsdale J. M. Fisher E. C. S. (2018). The importance of definitional and temporal issues in the study of resilience. *Appl. Psychol*. 68 583–620. 10.1111/apps.12162

[B94] Fletcher T. D. Major D. A. (2006). The effects of communication modality on performance and self-ratings of teamwork components. *J. Comput. Mediat. Commun*. 11 557–576. 10.1111/j.1083-6101.2006.00027.x

[B95] Ford K. J. Baldwin T. T. Prasad J. (2018). Transfer of training: the known and the unknown. *Annu. Rev. Organ. Psychol. Organ. Behav*. 5 201–225. 10.1146/annurev-orgpsych-032117-104443

[B96] Förster C. Duchek S. (2017). What makes leaders resilient? An exploratory interview study. *Ger. J. Hum. Resour. Manag*. 31 281–306. 10.1177/2397002217709400

[B97] Frey C. B. Osborne M. A. (2013). *The Future of Employment: How Susceptible are Jobs to Computerization?* Oxford: University of Oxford.

[B98] Gamrat C. Zimmerman H. T. Dudek J. Peck K. (2014). Personalized workplace learning: an exploratory study on digital badging within a teacher professional development program. *Br. J. Educ. Technol*. 45 1136–1148. 10.1111/bjet.12200

[B99] Gemeda H. K. Lee J. (2020). Leadership styles, work engagement and outcomes among information and communications technology professionals: a cross-national study. *Heliyon* 6:e03699. 10.1016/j.heliyon.2020.e03699 32280799 PMC7138911

[B100] Ghislieri C. Molino M. Cortese C. G. (2018). Work and organizational psychology looks at the fourth industrial revolution: how to support workers and organizations? *Front. Psychol* 9:2365. 10.3389/fpsyg.2018.02365 30546335 PMC6279953

[B101] Gibson C. B. (2001). From knowledge accumulation to accommodation: cycles of collective cognition in work groups. *J. Organ. Behav.* 22 121–134. 10.1002/job.84

[B102] Gorlitz K. Tamm M. (2016). Revisiting the complementarity between education and training: the role of job tasks and firm effects. *Educ. Econ.* 24 261–279. 10.1080/09645292.2015.1006182

[B103] Grant D. Newell S. (2013). Realizing the strategic potential of e-HRM. *J. Strateg. Inf. Syst.* 22 187–192. 10.1016/j.jsis.2013.07.001

[B104] Greenhalgh T. Robert G. Macfarlane F. Bate P. Kyriakidou O. Peacock R. (2005). Storylines of research in diffusion of innovation: a meta-narrative approach to systematic review. *Soc. Sci. Med.* 61 417–430. 10.1016/j.socscimed.2004.12.001 15893056

[B105] Greenhalgh T. Wherton J. Papoutsi C. Lynch J. Hughes G. A’Court C. (2017). Beyond adoption: a new framework for theorizing and evaluating nonadoption, abandonment, and challenges to the scale-up, spread, and sustainability of health and care technologies. *J. Med. Internet Res.* 19 e367. 10.2196/jmir.8775 29092808 PMC5688245

[B106] Grudin J. (2006). Enterprise knowledge management and emerging technologies. *Paper Presented at the 39th Annual Hawaii International Conference on System Sciences*, Los Alamitos, CA.

[B107] Grundke R. Marcolin L. Nguyen T. L. B. Squicciarini M. (2018). *Which skills for the digital era? Returns to skills analysis*. OECD Science, Technology and Industry Working Papers 2018/09 Paris: OECD Publishing, 10.1787/9a9479b5-en

[B108] Guinan P. J. Parise S. Langowitz N. (2019). Creating an innovative digital project team: levers to enable digital transformation. *Bus. Horiz.* 62 717–727. 10.1016/j.bushor.2019.07.005

[B109] Guo Y. U.-F. Cross W. Plummer V. Lam L. Luo Y.-H. Zhang J.-P. (2017). Exploring resilience in Chinese nurses: a cross-sectional study. *J. Nurs. Manag.* 25 223–230. 10.1111/jonm.12457 28164403

[B110] Gurtoo A. Tripathy A. (2000). Assessing workers’ attitude towards technological change: scale construction. *Indian J. Ind. Relat.* 35 519–531.

[B111] Haddad C. J. (1996). Employee attitudes toward new technology in a unionized manufacturing plant. *J. Eng. Technol. Manag.* 13 145–162. 10.1016/S0923-4748(96)01001-6

[B112] Haddud A. McAllen D. K. (2018). Digital workplace management: exploring aspects related to culture, innovation, and leadership. *Paper presented at the 2018 Portland International Conference on Management of Engineering and Technology (PICMET)*, Portland, OR.

[B113] Harms P. D. Vanhove A. Luthans F. (2017). Positive projections and health: an initial validation of the implicit psychological capital health measure. *Appl. Psychol.* 66 78–102. 10.1111/apps.12077

[B114] Harteis C. Goller M. (2014). “New skills for new jobs: work agency as a necessary condition for successful lifelong learning,” in *Promoting, Assessing, Recognizing and Certifying Lifelong Learning*, eds Billett S. Halttunen T. Koivisto M. (Dordrecht: Springer), 37–56.

[B115] Harter J. K. Schmidt F. L. Hayes T. L. (2002). Business-unit-level relationship between employee satisfaction, employee engagement, and business outcomes: a meta-analysis. *J. Appl. Psychol.* 87 268–279. 10.1037/0021-9010.87.2.268 12002955

[B116] Hartl E. Hess T. (2017). The role of cultural values for digital transformation: insights from a Delphi study. *Paper Presented at the 23rd Americas Conference on Information Systems*, Boston, MA.

[B117] Hartmann S. Weiss M. Newman A. Hoegl M. (2020). Resilience in the workplace: a multilevel review and synthesis. *Appl. Psychol.* 69 913–959. 10.1111/apps.12191

[B118] Hartnell C. A. Ou A. Y. Kinicki A. (2011). Organizational culture and organizational effectiveness: a meta-analytic investigation of the competing values framework’s theoretical suppositions. *J. Appl. Psychol.* 96 677–694. 10.1037/a0021987 21244127

[B119] Hausberg J. P. Liere-Netheler K. Packmohr S. Pakura S. Vogelsang K. (2019). Research streams on digital transformation from a holistic business perspective: a systematic literature review and citation network analysis. *J. Bus. Econ.* 89 931–963. 10.1007/s11573-019-00956-z

[B120] Hess T. Benlian A. Matt C. Wiesbock F. (2016). Options for formulating a digital transformation strategy. *MIS Q. Exec.* 15 123–139.

[B121] Hettich A. S. (2017). “Friend or foe? An exploratory analysis of employees’ attitudes towards self-service technologies,” in *Beiträge zur Dienstleistungsforschung*, ed. Büttgen M. (Wiesbaden: Springer Fachmedien Wiesbaden), 187–212.

[B122] Hong W. Thong J. Chasalow L. Dhillon G. (2011). User acceptance of agile information systems: a model and empirical test. *J. Manag. Inf. Syst.* 28 235–272. 10.2753/mis0742-1222280108

[B123] Huang J. L. Ryan A. M. Zabel K. L. Palmer A. (2014). Personality and adaptive performance at work: a meta-analytic investigation. *J. Appl. Psychol.* 99 162–179. 10.1037/a0034285 24016205

[B124] Huang L. V. Liu L. (2017). Ties that work: investigating the relationships among coworker connections, work-related Facebook utility, online social capital, and employee outcomes. *Comput. Hum. Behav.* 72 512–524. 10.1016/j.chb.2017.02.054

[B125] Huber G. P. (1990). A theory of the effects of advanced information technologies on organizational design, intelligence, and decision making. *Acad. Manag. Rev.* 15 47–71. 10.5465/amr.1990.4308227

[B126] Hur J.-Y. Cho W. Lee G. Bickerton S. H. (2019). The “smart work” myth: how bureaucratic inertia and workplace culture stymied digital transformation in the relocation of South Korea’s capital. *Asian Stud. Rev.* 43 691–709. 10.1080/10357823.2019.1663786

[B127] Janssen O. Huang X. (2008). Us and me: team identification and individual differentiation as complementary drivers of team members’ citizenship and creative behaviors. *J. Manag.* 34 69–88. 10.1177/0149206307309263

[B128] Jena R. K. (2015). Impact of technostress on job satisfaction: an empirical study among Indian academician. *Int. Technol. Manag. Rev.* 5 117–124. 10.2991/itmr.2015.5.3.1 32175718

[B129] Jensen P. M. Trollope-Kumar K. Waters H. Everson J. (2008). Building physician resilience. *Can. Fam. Physician.* 54 722–729. 18474706 PMC2377221

[B130] Johns G. (2018). Advances in the treatment of context in organizational research. *Annu. Rev. Organ. Psychol. Organ. Behav.* 5 21–46. 10.1146/annurev-orgpsych-032117-104406

[B131] Jones C. M. McCarthy R. Halawi L. (2010). Utilizing the technology acceptance model to assess the employee adoption of information systems security measures. *J. Intl. Technol. Inf. Manag.* 19 43–56.

[B132] Jones D. E. (1999). Ten years later: support staff perceptions and opinions on technology in the workplace. *Libr. Trends.* 47 711–745.

[B133] Jordan M. H. Feild H. S. Armenakis A. A. (2002). The relationship of group process variables and team performance. *Small Group Res.* 33 121–150. 10.1177/104649640203300104

[B134] Judge T. A. Thoresen C. J. Bono J. E. Patton G. K. (2001). The job satisfaction–job performance relationship: a qualitative and quantitative review. *Psychol. Bull.* 127 376–407. 10.1037/0033-2909.127.3.376 11393302

[B135] Jung D. I. Chow C. Wu A. (2003). The role of transformational leadership in enhancing organizational innovation: hypotheses and some preliminary findings. *Leadersh. Q.* 14 525–544. 10.1016/S1048-9843(03)00050-X

[B136] Jung H. S. Yoon H. H. (2015). The impact of employees’ positive psychological capital on job satisfaction and organizational citizenship behaviors in the hotel. *Int. J. Contemp. Hosp. Manag.* 27 1135–1156. 10.1108/IJCHM-01-2014-0019

[B137] Kaasinen E. Liinasuo M. Schmalfuß F. Koskinen H. Aromaa S. Heikkilä P. (2018). “A worker-centric design and evaluation framework for operator 4.0 solutions that support work well-being,” in *Proceedings of the IFIP Working Conference on Human Work Interaction Design* (Cham: Springer), 263–282. 10.1007/978-3-030-05297-3_18

[B138] Kagan J. (2016). An overly permissive extension. *Perspect. Psychol. Sci.* 11 442–450. 10.1177/1745691616635593 27474132

[B139] Kane G. C. Palmer D. Phillips A. N. Kiron D. Buckley N. (2015). *Strategy, Not Technology, Drives Digital Transformation.* Available online at: https://sloanreview.mit.edu/projects/strategy-drives-digital-transformation/ (accessed October 10, 2020).

[B140] Karacay G. (2018). *“Talent development for industry 4.0,” in Industry 4.0: Managing the Digital Transformation.* Cham: Springer International Publishing, 123–136.

[B141] Karimi J. Walter Z. (2015). The role of dynamic capabilities in responding to digital disruption: a factor-based study of the newspaper industry. *J. Manag. Inf. Syst.* 32 39–81. 10.1080/07421222.2015.1029380

[B142] Kazmi R. Amjad S. Khan D. (2008). Occupational stress and its effect on job performance: a case study of medical house officers of district Abbottabad. *J. Ayub. Med. Coll. Abbottabad.* 20 135–139. 19610539

[B143] Kim C. Jahng J. Lee J. (2007). An empirical investigation into the utilization-based information technology success model: integrating task-performance and social influence perspective. *J. Inf. Technol.* 22 152–160. 10.1057/palgrave.jit.2000072

[B144] Kim H.-W. Kankanhalli A. (2009). Investigating user resistance to information systems implementation: a status quo bias perspective. *Manag. Inf. Syst. Q.* 33 567–582. 10.2307/20650309

[B145] Kim M.-K. Joo C. Park J.-H. (2017). Investigating the determinants of low adoption of tablet PCs in Korean firms: effects of value perception and alternative attractiveness. *Telemat. Inform.* 34 1557–1571. 10.1016/j.tele.2017.07.003

[B146] Kim Y. Kim S.-S. (2018). Job insecurity and depression among automobile sales workers: a longitudinal study in South Korea. *Am. J. Ind. Med*. 61 140–147. 10.1002/ajim.22805 29226347

[B147] King W. R. He J. (2006). A meta-analysis of the technology acceptance model. *Inf. Manag.* 43 740–755. 10.1016/j.im.2006.05.003

[B148] Kinicki A. J. McKee-Ryan F. M. Schriesheim C. A. Carson K. P. (2002). Assessing the construct validity of the job descriptive index: a review and meta-analysis. *J. Appl. Psychol.* 87 14–32. 10.1037/0021-9010.87.1.14 11916208

[B149] Kinman G. Grant L. (2011). Exploring stress resilience in trainee social workers: the role of emotional and social competencies. *Br. J. Soc. Work.* 41 261–275. 10.1093/bjsw/bcq088

[B150] Kirkman B. L. Mathieu J. E. (2005). The dimensions and antecedents of team virtuality. *J. Manag.* 31 700–718. 10.1177/0149206305279113

[B151] Klein K. J. Conn A. B. Smith D. B. Sorra J. S. (2001). Is everyone in agreement? An exploration of within-group agreement in employee perceptions of the work environment. *J. Appl. Psychol.* 86 3–16. 10.1037/0021-9010.86.1.3 11302231

[B152] Kossek E. E. Perrigino M. B. (2016). Resilience: a review using a grounded integrated occupational approach. *Acad. Manag. Ann.* 10 729–797. 10.1080/19416520.2016.1159878

[B153] Koys D. J. (2001). The effects of employee satisfaction, organizational citizenship behavior, and turnover on organizational effectiveness: a unit-level, longitudinal study. *Pers. Psychol.* 54 101–114. 10.1111/j.1744-6570.2001.tb00087.x

[B154] Kozlowski S. W. J. Ilgen D. R. (2006). Enhancing the effectiveness of work groups and teams. *Psychol. Sci. Public Interest* 7 77–124. 10.1111/j.1529-1006.2006.00030.x 26158912

[B155] Kozlowski S. W. J. Klein K. J. (2000). “A multilevel approach to theory and research in organizations: contextual, temporal, and emergent processes,” in *Multilevel Theory, Research, and Methods in Organizations: Foundations, Extensions, and New Directions*, eds Klein K. J. Kozlowski S. W. J. (San Francisco, CA: Jossey-Bas), 3–90.

[B156] Krekel C. Ward G. Neve J.-E. (2019). Employee wellbeing, productivity, and firm performance. *SSRN Electron. J.* Available online at: https://voxeu.org/article/employee-wellbeing-productivity-and-firm-performance (accessed October 10, 2020).

[B157] Lam T. Cho V. Qu H. (2007). A study of hotel employee behavioral intentions towards adoption of information technology. *Int. J. Hosp. Manag*. 26 49–65. 10.1016/j.ijhm.2005.09.002

[B158] Lamb D. Cogan N. (2016). Coping with work-related stressors and building resilience in mental health workers: A comparative focus group study using interpretative phenomenological analysis. *J. Occup. Organ. Psychol*. 89 474–492. 10.1111/joop.12136

[B159] Larson M. Luthans F. (2006). Potential added value of psychological capital in predicting work attitudes. *J. Leadersh. Organ. Stud.* 13 45–62. 10.1177/10717919070130010701

[B160] Lee Y. Kozar K. A. Larsen K. (2003). The technology acceptance model: past, present, and future. *Commun. Assoc. Inf. Syst.* 12 752–780. 10.17705/1CAIS.01250

[B161] Lee Y.-H. Hsieh Y.-C. Chen Y.-H. (2013). An investigation of employees’ use of e-learning systems: applying the technology acceptance model. *Behav. Inf. Technol.* 32 173–189. 10.1080/0144929X.2011.577190

[B162] Leonardi P. M. Huysman M. Steinfield C. (2013). Enterprise social media: definition, history, and prospects for the study of social technologies in organizations. *J. Comput. Mediat. Commun.* 19 1–19. 10.1111/jcc4.12029

[B163] Lepine J. A. Podsakoff N. P. Lepine M. A. (2005). A meta-analytic test of the challenge stressor-hindrance stressor framework: an explanation for inconsistent relationships among stressors and performance. *Acad. Manag. J.* 48 764–775. 10.2307/20159696

[B164] Li J. Bonn M. A. Ye H. (2019). Hotel employee’s artificial intelligence and robotics awareness and its impact on turnover intention: the moderating roles of perceived organizational support and competitive psychological climate. *Tour. Manag.* 73 172–181. 10.1016/j.tourman.2019.02.006

[B165] Li J. Herd A. M. (2017). Shifting practices in digital workplace learning: an integrated approach to learning, knowledge management, and knowledge sharing. *Hum. Resour. Dev. Int.* 20 185–193. 10.1080/13678868.2017.1308460

[B166] Li L. Su F. Zhang W. Mao J.-Y. (2018). Digital transformation by SME entrepreneurs: a capability perspective. *Inf. Syst. J.* 28 1129–1157. 10.1111/isj.12153

[B167] Liao H. Liu D. Loi R. (2010). Looking at both sides of the social exchange coin: a social cognitive perspective on the joint effects of relationship quality and differentiation on creativity. *Acad. Manag.* J. 53 1090–1109. 10.5465/AMJ.2010.54533207

[B168] Liao Y. Deschamps F. de Freitas E. Ramos L. F. P. (2017). Past, present and future of Industry 4.0: a systematic literature review and research agenda proposal. *Int. J. Prod. Res.* 55 3609–3629. 10.1080/00207543.2017.1308576

[B169] Liao Z. Landry R. (2000). An empirical study on organizational acceptance of new information systems in a commercial bank environment. *Paper Presented at the 33rd Annual Hawaii International Conference on System Sciences*, Maui, HI.

[B170] Liu C.-F. Cheng T.-J. Chen C.-T. (2019). Exploring the factors that influence physician technostress from using mobile electronic medical records. *Inform. Health Soc. Care* 44 92–104. 10.1080/17538157.2017.1364250 29068768

[B171] Lloréns-Montes F. J. Ruiz-Moreno A. Garcıìa-Morales V. (2005). Influence of support leadership and teamwork cohesion on organizational learning, innovation and performance: an empirical examination. *Technovation* 25 1159–1172. 10.1016/j.technovation.2004.05.002

[B172] Lounsbury J. W. Loveland J. M. Sundstrom E. D. Gibson L. W. Drost A. W. Hamrick F. L. (2003). An investigation of personality traits in relation to career satisfaction. *J. Career Assess.* 11 287–307. 10.1177/1069072703254501

[B173] Luthans F. Avolio B. J. Avey J. B. Norman S. M. (2007). Positive psychological capital: measurement and relationship with performance and satisfaction. *Pers. Psychol.* 60 541–572. 10.1111/j.1744-6570.2007.00083.x

[B174] Luthans F. Avolio B. J. Walumbwa F. O. Li W. (2005). The psychological capital of Chinese workers: exploring the relationship with performance. *Manag. Organ. Rev.* 1 249–271. 10.1111/j.1740-8784.2005.00011.x

[B175] Luthar S. S. Cicchetti D. Becker B. (2000). The construct of resilience: a critical evaluation and guidelines for future work. *Child Dev.* 71 543–562. 10.1111/1467-8624.00164 10953923 PMC1885202

[B176] Lyons S. T. Schweitzer L. Ng E. S. W. (2015). Resilience in the modern career. *Career Dev. Int.* 20 363–383. 10.1108/CDI-02-2015-0024

[B177] Mache S. Vitzthum K. Wanke E. Patterson D. A. Klapp B. F. Danzer G. (2014). Exploring the impact of resilience, self-efficacy, optimism and organizational resources on work engagement. *Work* 47 491–500. 10.3233/wor-131617 23531578

[B178] Malik P. Garg P. (2017). The relationship between learning culture, inquiry and dialogue, knowledge sharing structure and affective commitment to change. *J. Organ. Chang. Manag.* 30 610–631. 10.1108/JOCM-09-2016-0176

[B179] Marks M. A. Mathieu J. E. Zaccaro S. J. (2001). A temporally based framework and taxonomy of team processes. *Acad. Manag. Rev.* 26 356–376. 10.2307/259182

[B180] Marler J. H. Boudreau J. W. (2017). An evidence-based review of HR analytics. *Int. J. Hum. Resour. Manag.* 28 3–26. 10.1080/09585192.2016.1244699

[B181] Marler J. H. Fisher S. L. (2013). An evidence-based review of e-HRM and strategic human resource management. *Hum. Resour. Manag. Rev*. 23 18–36. 10.1016/j.hrmr.2012.06.002

[B182] Marlow S. L. Lacerenza C. N. Paoletti J. Burke C. S. Salas E. (2018). Does team communication represent a one-size-fits-all approach: A meta-analysis of team communication and performance. *Organ. Behav. Hum. Decis. Process.* 144 145–170. 10.1016/j.obhdp.2017.08.001

[B183] Martín-Rojas R. García-Morales V. J. González-Álvarez N. (2019). Technological antecedents of entrepreneurship and its consequences for organizational performance. *Technol. Forecast. Soc. Change.* 147 22–35. 10.1016/j.techfore.2019.06.018

[B184] Matt C. Hess T. Benlian A. (2015). Digital transformation strategies. *Bus. Inf. Syst. Eng.* 57 339–343. 10.1007/s12599-015-0401-5

[B185] McDonald G. Jackson D. Vickers M. H. Wilkes L. (2016). Surviving workplace adversity: a qualitative study of nurses and midwives and their strategies to increase personal resilience. *J. Nurs. Manag.* 24 123–131. 10.1111/jonm.12293 25865519

[B186] McKinsey & Company (2017). *Jobs Lost, Jobs Gained: Workforce Transitions in a Time of Automation.* Available online at https://www.mckinsey.com/featured-insights/future-of-work/jobs-lost-jobs-gained-what-the-future-of-work-will-mean-for-jobs-skills-and-wages (accessed October 10, 2020).

[B187] McKinsey (2021). *Five Fifty: The skillful corporaton.* Available online at https://www.mckinsey.com/business-functions/mckinsey-accelerate/our-insights/five-fifty-the-skillful-corporation

[B188] Melián-González S. Bulchand-Gidumal J. (2017). Information technology and front office employees’ performance. *Int. J. Contemp. Hosp. Manag.* 29 2159–2177. 10.1108/IJCHM-10-2015-0585

[B189] Meneghel I. Martínez M. I. Salanova M. (2016a). Job-related antecedents of team resilience and improved team performance. *Pers. Rev*. 45 505–522. 10.1108/PR-04-2014-0094

[B190] Meneghel I. Salanova M. Martínez I. M. (2016b). Feeling good makes us stronger: how team resilience mediates the effect of positive emotions on team performance. *J. Happiness Stud.* 17 239–255. 10.1007/s10902-014-9592-6

[B191] Mercader C. Gairín J. (2020). University teachers’ perception of barriers to the use of digital technologies: the importance of the academic discipline. *Int. J. Educ. Technol. High. Educ.* 17 1–14. 10.1186/s41239-020-0182-x

[B192] Merschbrock C. Munkvold B. E. (2015). Effective digital collaboration in the construction industry: a case study of BIM deployment in a hospital construction project. *Comput. Ind.* 73 1–7. 10.1016/j.compind.2015.07.003

[B193] Meske C. Junglas I. (2020). Investigating the elicitation of employees’ support towards digital workplace transformation. *Behav. Inf. Technol.* 39 1–17. 10.1080/0144929x.2020.1742382

[B194] Mesmer-Magnus J. R. DeChurch L. A. (2009). Information sharing and team performance: a meta-analysis. *J. Appl. Psychol.* 94 535–546. 10.1037/a0013773 19271807

[B195] Molino M. Cortese C. G. Ghislieri C. (2020). The promotion of technology acceptance and work engagement in industry 4.0: from personal resources to information and training. *Int. J. Environ. Res. Public Health* 17:2438. 10.3390/ijerph17072438 32260142 PMC7178190

[B196] Mueller B. Renken U. (2017). Helping employees to be digital transformers: the Olympus.connect case. *Paper Presented at the 38th International Conference on Information Systems*, Seoul.

[B197] Mukherji S. Arora N. (2017). “Digital communication: easing operational outcomes in the workplace,” in *Proceedings of the 5th International Conference on Information Technology and Quantitative Management*, eds Ahuja V. Shi Y. Khazanchi D. Abidi N. Tian Y. Berg D. (Amsterdam: Elsevie), 1084–1091.

[B198] Nam C. S. Lyons J. B. Hwang H.-S. Kim S. (2009). The process of team communication in multi-cultural contexts: an empirical study using Bales’ interaction process analysis (IPA). *Int. J. Ind. Ergon.* 39 771–782. 10.1016/j.ergon.2009.03.004

[B199] Niedzwiecka M. Pan Y.-C. (2017). An exploratory study into employee attitudes towards digitalization of library services in higher education. *Paper Presented at the 22nd UK Academy for Information Systems Annual Conference (UKAIS 2017)*, Oxford.

[B200] Nisafani A. S. Kiely G. Mahony C. (2020). Workers’ technostress: a review of its causes, strains, inhibitors, and impacts. *J. Decis. Syst.* 1–16. 10.1080/12460125.2020.1796286

[B201] Noe R. A. Clarke A. D. M. Klein H. J. (2014). Learning in the twenty-first-century workplace. *Annu. Rev. Organ. Psychol. Organ. Behav.* 1 245–275. 10.1146/annurev-orgpsych-031413-091321

[B202] Oberlander M. Beinicke A. Bipp T. (2020). Digital competencies: a review of the literature and applications in the workplace. *Comput. Educ.* 146:103752. 10.1016/j.compedu.2019.103752

[B203] Orlikowski W. J. (1992). The duality of technology: rethinking the concept of technology in organizations. *Organ. Sci.* 3 398–427. 10.1287/orsc.3.3.398 19642375

[B204] Orlikowski W. J. (2010). The sociomateriality of organisational life: considering technology in management research. *Camb. J. Econ.* 34 125–141. 10.1093/cje/bep058

[B205] Osmundsen K. Iden J. Bygstad B. (2018). Digital transformation drivers, success factors, and implications. *Paper Presented at the 12th Mediterranean Conference on Information Systems*, Korfu.

[B206] Osmundsen K. S. (2020). Competences for digital transformation: insights from the Norwegian energy sector. *Paper Presented at the 53rd Hawaii International Conference on System Sciences*, Maui, HI.

[B207] Ostroff C. Kinicki A. Tamkins M. M. (2003). “Organizational culture and climate,” in *Handbook of Psychology*, eds Borman W. C. Ilgen D. R. Klimoski R. J. (Hoboken, NJ: John Wiley & Sons, Inc), 565–593.

[B208] Patterson M. G. West M. A. Shackleton V. J. Dawson J. F. Lawthom R. Maitlis S. (2005). Validating the organizational climate measure: links to managerial practices, productivity and innovation. *J. Organ. Behav.* 26 379–408. 10.1002/job.312

[B209] Ployhart R. E. (2012). The psychology of competitive advantage: an adjacent possibility. *Ind. Organ. Psychol.* 5 62–81. 10.1111/j.1754-9434.2011.01407.x

[B210] Ployhart R. E. (2015). Strategic organizational behavior (STROBE): the missing voice in the strategic human capital conversation. *Acad. Manag. Perspect.* 29 342–356. 10.5465/amp.2014.0145

[B211] Ployhart R. E. Bliese P. D. (2006). “Individual adaptability (I-ADAPT) theory: conceptualizing the antecedents, consequences, and measurement of individual differences in adaptability,” in *Understanding Adaptability: A Prerequisite for Effective Performance Within Complex Environments*, eds Salas E. Pierce L. G. Shawn Burke C. (Amsterdam: Elsevier), 3–39.

[B212] Pulakos E. D. Dorsey D. W. White S. S. (2006). “Adaptability in the workplace: selecting an adaptive workforce,” in *Understanding Adaptability: A Prerequisite for Effective Performance With in Complex Environments*, eds Burke S. C. Pierce L. G. Salas E. (Amsterdam: Elsevier), 41–71.

[B213] Quinn R. E. Rohrbaugh J. (1983). A spatial model of effectiveness criteria: towards a competing values approach to organizational analysis. *Manag. Sci*. 29 363–377.

[B214] Ragu-Nathan T. S. Tarafdar M. Ragu-Nathan B. S. Tu Q. (2008). The consequences of technostress for end users in organizations: conceptual development and empirical validation. *Inf. Syst. Res.* 19 417–433. 10.1287/isre.1070.0165 19642375

[B215] Riketta M. (2008). The causal relation between job attitudes and performance: a meta-analysis of panel studies. *J. Appl. Psychol.* 93 472–481. 10.1037/0021-9010.93.2.472 18361647

[B216] Robbins S. P. Judge T. A. (2019). *Organizational Behavior*, 18th Edn. New York, NY: Pearson.

[B217] Roepke R. Agarwal R. Ferratt T. W. (2000). Aligning the IT human resource with business vision: the leadership initiative at 3M. *Manag. Inf. Syst. Q.* 24 327–353. 10.2307/3250941

[B218] Rogers E. (1995). *Diffusion of Innovations*, 4th Edn. New York, NY: The Free Press.

[B219] Sanders K. Moorkamp M. Torka N. Groeneveld S. Groeneveld C. (2010). How to support innovative behavior? The role of LMX and satisfaction with HR practices. *Technol. Invest*. 1 59–68. 10.4236/ti.2010.11007

[B220] Sarwar S. Dent A. Faust K. Richer M. Djuric U. Van Ommeren R. (2019). Physician perspectives on integration of artificial intelligence into diagnostic pathology. *NPJ Digit. Med.* 2:28. 10.1038/s41746-019-0106-0 31304375 PMC6550202

[B221] Schein E. H. (2004). *Organizational Culture and Leadership.* San Fransisco, CA: Wiley.

[B222] Schlagwein D. Hu M. (2017). How and why organisations use social media: five use types and their relation to absorptive capacity. *J. Inf. Technol.* 32 194–209. 10.1057/jit.2016.7

[B223] Schneider B. Ehrhart M. G. Macey W. H. (2013). Organizational climate and culture. *Annu. Rev. Psychol.* 64 361–388. 10.1146/annurev-psych-113011-143809 22856467

[B224] Schneider B. Hanges P. J. Smith D. B. Salvaggio A. N. (2003). Which comes first: employee attitudes or organizational financial and market performance? *J. Appl. Psychol.* 88 836–851. 10.1037/0021-9010.88.5.836 14516248

[B225] Schraeder M. Swamidass P. M. Morrison R. (2006). Employee involvement, attitudes and reactions to technology changes. *J. Leadersh. Organ. Stud.* 12 85–100. 10.1177/107179190601200306

[B226] Schwab K. (2015). *The Fourth Industrial Revolution: What it Means and How to Respond.* Available online at: https://www.foreignaffairs.com/articles/2015-12-12/fourth-industrial-revolution (accessed October 10, 2020).

[B227] Sebastian I. M. Moloney K. G. Ross J. W. Fonstad N. O. Beath C. Mocker M. (2017). How big old companies navigate digital transformation. *MIS Q. Exec.* 16 197–213. 10.4324/9780429286797-6

[B228] Seers A. Petty M. M. Cashman J. F. (1995). Team-member exchange under team and traditional management: a naturally occurring quasi-experiment. *Group Organ. Manag.* 20 18–38. 10.1177/1059601195201003

[B229] Seligman M. E. P. Csikszentmihalyi M. (2000). Positive psychology: an introduction. *Am. Psychol.* 55 5–14. 10.1037/0003-066X.55.1.5 11392865

[B230] Shanker R. Bhanugopan R. van der Heijden B. I. J. M. Farrell M. (2017). Organizational climate for innovation and organizational performance: the mediating effect of innovative work behavior. *J. Vocat. Behav*. 100 67–77. 10.1016/j.jvb.2017.02.004

[B231] Shipton H. Fay D. West M. Patterson M. Birdi K. (2005). Managing people to promote innovation. *Creat. Innov. Manag.* 14 118–128. 10.1111/j.1467-8691.2005.00332.x

[B232] Sias P. M. (2009). *Organizing Relationships: Traditional and Emerging Perspectives on Workplace Relationships.* Thousand Oaks, CA: Sage.

[B233] Sias P. M. Duncan K. L. (2018). Not just for customers anymore: organization Facebook, employee social capital, and organizational identification. *Int. J. Bus. Commun*. 45 1–21. 10.1177/2329488418765930

[B234] Sias P. M. Perry T. (2004). Disengaging from workplace relationships: a research note. *Hum. Commun. Res*. 30 589–602. 10.1111/j.1468-2958.2004.tb00746.x

[B235] Silvestro R. (2002). Dispelling the modern myth: employee satisfaction and loyalty drive service profitability. *Int. J. Oper. Prod. Manag*. 22 30–49. 10.1108/01443570210412060

[B236] Skog D. A. Wimelius H. Sandberg J. (2018). Digital disruption. *Bus. Inf. Syst. Eng.* 60 431–437. 10.1007/s12599-018-0550-4

[B237] Son H. Park Y. Kim C. Chou J.-S. (2012). Toward an understanding of construction professionals’ acceptance of mobile computing devices in South Korea: an extension of the technology acceptance model. *Autom. Constr.* 28 82–90. 10.1016/j.autcon.2012.07.002

[B238] Sousa M. J. Rocha Á (2019). Digital learning: developing skills for digital transformation of organizations. *Future Gener. Comput. Syst* 91 327–334. 10.1016/j.future.2018.08.048

[B239] Stephens J. P. Heaphy E. D. Carmeli A. Spreitzer G. M. Dutton J. E. (2013). Relationship quality and virtuousness: emotional carrying capacity as a source of individual and team resilience. *J. Appl. Behav. Sci*. 49 13–41. 10.1177/0021886312471193

[B240] Stevenson A. D. Phillips C. B. Anderson K. J. (2011). Resilience among doctors who work in challenging areas: a qualitative study. *Br. J. Gen. Pract.* 61 e404–e410. 10.3399/bjgp11X583182 21722448 PMC3123503

[B241] Stoverink A. C. Kirkman B. L. Mistry S. Rosen B. (2018). Bouncing back together: toward a theoretical model of work team resilience. *Acad. Manag. Rev.* 45 395–422. 10.5465/amr.2017.0005

[B242] Talukder M. (2012). Factors affecting the adoption of technological innovation by individual employees: an Australian study. *Proc. Soc. Behav. Sci.* 40 52–57. 10.1016/j.sbspro.2012.03.160

[B243] Tarafdar M. Cooper C. L. Stich J.-F. (2019). The technostress trifecta: techno eustress, techno distress and design: theoretical directions and an agenda for research. *Inf. Syst. J.* 29 6–42. 10.1111/isj.12169

[B244] Tarafdar M. Pullins E. B. Ragu-Nathan T. S. (2015). Technostress: negative effect on performance and possible mitigations. *Inf. Syst. J.* 25 103–132. 10.1111/isj.12042

[B245] Tarafdar M. Tu Q. Ragu-Nathan T. S. (2010). Impact of technostress on end-user satisfaction and performance. *J. Manag. Inf. Syst.* 27 303–334. 10.2753/MIS0742-1222270311

[B246] Tasdogan A. M. (2020). Knowledge, attitudes and perspectives of anesthesiologists on artificial intelligence. *Eurasian J. Med. Invest*. 4 1–6. 10.14744/ejmi.2020.54709

[B247] Tekic Z. Koroteev D. (2019). From disruptively digital to proudly analog: a holistic typology of digital transformation strategies. *Bus. Horiz.* 62 683–693. 10.1016/j.bushor.2019.07.002

[B248] Tenney E. R. Logg J. M. Moore D. A. (2015). (Too) optimistic about optimism: the belief that optimism improves performance. *J. Pers. Soc. Psychol.* 108 377–399. 10.1037/pspa0000018 25751715

[B249] Tenney E. R. Poole J. M. Diener E. (2016). Does positivity enhance work performance: why, when, and what we don’t know. *Res. Organ. Behav*. 36 27–46. 10.1016/j.riob.2016.11.002

[B250] Treem J. W. Leonardi P. M. (2012). Social media use in organizations: exploring the affordances of visibility, editablity, persistence, and association. *Commun. Yearb*. 36 143–189. 10.2139/ssrn.2129853

[B251] Tripsas M. (2009). Technology, identity, and inertia through the lens of ‘the digital photography company”. *Organ. Sci.* 20 441–460. 10.1287/orsc.1080.0419 19642375

[B252] Tyworth M. (2014). Organizational identity and information systems: how organizational ICT reflect who an organization is. *Eur. J. Inf. Syst.* 23 69–83. 10.1057/ejis.2013.32

[B253] Utesheva A. Simpson J. R. Cecez-Kecmanovic D. (2016). Identity metamorphoses in digital disruption: a relational theory of identity. *Eur. J. Inf. Syst*. 25 344–363. 10.1057/ejis.2015.19

[B254] Vallerand R. J. (1997). “Toward a hierarchical model of intrinsic and extrinsic motivation,” in *Advances in Experimental Social Psychology*, ed. Zanna M. P. (Cambridge, MA: Academic Press), 271–360.

[B255] van Der Vegt G. S. Bunderson J. S. (2005). Learning and performance in multidisciplinary teams: the importance of collective team identification. *Acad. Manag. J.* 43 532–547. 10.2307/20159674

[B256] Veiga J. F. Keupp M. M. Floyd S. W. Kellermanns F. W. (2014). The longitudinal impact of enterprise system users’ pre-adoption expectations and organizational support on post-adoption proficient usage. *Eur. J. Inf. Syst*. 23 691–707. 10.1057/ejis.2013.15

[B257] Venkatesh V. (2000). Determinants of perceived ease of use: integrating control, intrinsic motivation, and emotion into the technology acceptance model. *Inf. Syst. Res.* 11 342–365. 10.1287/isre.11.4.342.11872 19642375

[B258] Venkatesh V. (2006). Where to go from here? Thoughts on future directions for research on individual-level technology adoption with a focus on decision making^∗^. *Decis. Sci*. 37 497–518. 10.1111/j.1540-5414.2006.00136.x

[B259] Venkatesh V. Bala H. (2008). Technology acceptance model 3 and a research agenda on interventions. *Decis. Sci.* 31 273–315. 10.1111/j.1540-5915.2008.00192.x

[B260] Venkatesh V. Davis F. D. (2000). A theoretical extension of the technology acceptance model: four longitudinal field studies. *Manag. Sci.* 46 186–204. 10.1287/mnsc.46.2.186.11926 19642375

[B261] Venkatesh V. Morris M. G. Davis G. B. Davis F. D. (2003). User acceptance of information technology: toward a unified view. *Manag. Inf. Syst. Q*. 27 425–478. 10.2307/30036540

[B262] Venkatesh V. Thong J. Xu X. (2016). Unified theory of acceptance and use of technology: a synthesis and the road ahead. *J. Assoc. Inf. Syst*. 17 328–376. 10.17705/1jais.00428

[B263] Verhoef P. C. Broekhuizen T. Bart Y. Bhattacharya A. Qi Dong J. Fabian N. (2019). Digital transformation: a multidisciplinary reflection and research agenda. *J. Bus. Res.* 122 889–901. 10.1016/j.jbusres.2019.09.022

[B264] Vial G. (2019). Understanding digital transformation: a review and a research agenda. *J. Strateg. Inf. Syst* 28 118–144. 10.1016/j.jsis.2019.01.003

[B265] Vieitez J. C. Carcía A. D. L. T. Rodríguez M. T. V. (2001). Perception of job security in a process of technological change: its influence on psychological well-being. *Behav. Inf. Technol.* 20 213–223. 10.1080/01449290120718

[B266] Wanberg C. R. Banas J. T. (2000). Predictors and outcomes of openness to changes in a reorganizing workplace. *J. Appl. Psychol.* 85 132–142. 10.1037/0021-9010.85.1.132 10740964

[B267] Wang T. Jung C.-H. Kang M.-H. Chung Y.-S. (2014). Exploring determinants of adoption intentions towards Enterprise 2.0 applications: an empirical study. *Behav. Inf. Technol.* 33 1048–1064. 10.1080/0144929X.2013.781221

[B268] Wagner J. A. Hollenbeck J. R. (2010). *Organizational Behavior: Securing Competitive Advantage.* Oxfordshire: Routledge.

[B269] Warner K. S. R. Wäger M. (2019). Building dynamic capabilities for digital transformation: an ongoing process of strategic renewal. *Long Range Plann.* 52 326–349. 10.1016/j.lrp.2018.12.001

[B270] Webster J. Watson R. (2002). Analyzing the past to prepare for the future: writing a literature review. *MIS Q.* 26 13–23. 10.2307/4132319

[B271] Wei W. Taormina R. J. (2014). A new multidimensional measure of personal resilience and its use: Chinese nurse resilience, organizational socialization and career success. *Nurs. Inq.* 21 346–357. 10.1111/nin.12067 24707977

[B272] Weick K. E. Quinn R. E. (1999). Organizational change and development. *Annu. Rev. Psychol.* 50 361–386. 10.1146/annurev.psych.50.1.361 15012461

[B273] Welbourne J. L. Gangadharan A. Sariol A. M. (2015). Ethnicity and cultural values as predictors of the occurrence and impact of experienced workplace incivility. *J. Occup. Health Psychol*. 20 205–217. 10.1037/a0038277 25365631

[B274] Wessel L. Baiyere A. Ologeanu-Taddei R. Cha J. Blegind Jensen T. (2020). Unpacking the difference between digital transformation and IT-enabled organizational transformation. *J. Assoc. Inf. Syst.* 22:6.

[B275] Westerman G. Bonnet D. McAfee A. (2014). *The Nine Elements of Digital Transformation. MIT Sloan Manage Reiewv.* Available online at: https://sloanreview.mit.edu/article/the-nine-elements-of-digital-transformation/ (accessed October 10, 2020).

[B276] World Economic Forum (2020). *The Future of Jobs Report.* Available online at: http://www3.weforum.org/docs/WEF_Future_of_Jobs_2020.pdf (accessed February 15, 2020).

[B277] Wright T. A. Cropanzano R. Bonett D. G. (2007). The moderating role of employee positive well being on the relation between job satisfaction and job performance. *J. Occup. Health Psychol.* 12 93–104. 10.1037/1076-8998.12.2.93 17469992

[B278] Wright T. A. Cropanzano R. Denney P. J. Moline G. L. (2002). When a happy worker is a productive worker: a preliminary examination of three models. *Can. J. Behav. Sci.* 34 146–150. 10.1037/h0087165

[B279] Yanez Morales V. P. Pan A. Ali U. (2020). How helping behaviours at work stimulate innovation in the organization: evidence from a moderated-mediation model. *Innovation* 22 71–90. 10.1080/14479338.2019.1632712

[B280] Yang Y. Danes S. M. (2015). Resiliency and resilience process of trepreneurs in new venture creation. *Entrep. Res. J*. 5 1–30. 10.1515/erj-2013-0076

[B281] Youssef C. M. Luthans F. (2007). Positive organizational behavior in the workplace: the impact of hope, optimism, and resilience. *J. Manag*. 33 774–800. 10.1177/0149206307305562

[B282] Yukl G. A. (2006). *Leadership in Organizations.* Upper Saddle River, NJ: Pearson/Prentice Hall.

[B283] Zaccaro S. J. Klimoski R. (2002). The interface of leadership and team processes. *Group Organ. Manag*. 27 4–13. 10.1177/1059601102027001002

[B284] Zeike S. Bradbury K. Lindert L. Pfaff H. (2019a). Digital leadership skills and associations with psychological well-being. *Int. J. Environ. Res. Public Health.* 16 1–12. 10.3390/ijerph16142628 31340579 PMC6678159

[B285] Zeike S. Choi K.-E. Lindert L. Pfaff H. (2019b). Managers’ well-being in the digital era: is it associated with perceived choice overload and pressure from digitalization? An exploratory study. *Int. J. Environ. Res. Public Health.* 16 1–15. 10.3390/ijerph16101746 31108843 PMC6572357

[B286] Zohar D. M. Hofmann D. A. (2012). “Organizational culture and climate,” in *The Oxford Handbook of Organizational Psychology*, ed. Kozlowski S. W. J. (Oxford: Oxford University Press), 643–666.

